# Dual HER2 Blockade versus a Single Agent in Trastuzumab-Containing Regimens for HER2-Positive Early Breast Cancer: A Systematic Review and Meta-Analysis of Randomized Controlled Trials

**DOI:** 10.1155/2020/5169278

**Published:** 2020-03-16

**Authors:** Liuwen Yu, Fangmeng Fu, Jing Li, Meng Huang, Bangwei Zeng, Yuxiang Lin, Qian Mei, Jinxing Lv, Chuan Wang

**Affiliations:** ^1^Breast Surgery Ward, Department of General Surgery, Fujian Medical University Union Hospital, Fuzhou, Fujian Province, China; ^2^Fujian Center for Disease Control and Prevention, Fuzhou, Fujian Province, China; ^3^Nosocomial Infection Control Branch, Fujian Medical University Union Hospital, Fuzhou, Fujian Province, China

## Abstract

**Purpose:**

Although trastuzumab is the standard of care for patients with human epidermal growth factor receptor 2 (HER2)- positive early breast cancer (EBC), drug resistance and disease relapse occur. Therefore, we performed a meta-analysis to assess the efficacy and safety of trastuzumab-containing dual anti-HER2 therapy compared to trastuzumab alone.

**Methods:**

A systematic search was performed to identify eligible randomized controlled trials (RCTs). Main outcomes including event-free survival/invasive disease-free survival (EFS/iDFS), overall survival (OS), and safety were considered.

**Results:**

Ten RCTs were included (15,284 patients). Significant improvements were observed in both EFS/iDFS (HR 0.86, *p*=0.0003) and OS (HR 0.86, *p*=0.02) with trastuzumab-based dual anti-HER2 therapy, especially in adjuvant treatment, while in the neoadjuvant setting, dual-targeted therapy also achieved a substantial pathological complete response (pCR) benefit (HR 1.34, *p*=0.0002). Subgroup analysis revealed that the EFS/iDFS benefit was slightly higher with trastuzumab plus pertuzumab or plus neratinib than trastuzumab plus lapatinib, while OS benefit was significant with trastuzumab plus lapatinib, but there were no subgroup differences (interaction test, *p*=0.80 and 0.24, resp.). In addition, EFS/iDFS benefit was unrelated to hormone receptor status but pronounced in the lymph node-positive (LN+) subgroup, which should be interpreted cautiously for lacking interaction (*p*=0.18). Besides, patients receiving dual therapy, especially with the lapatinib-containing regimen, experienced more toxicity, but no increase in cardiotoxicity.

**Conclusions:**

Despite being associated with more toxicity, trastuzumab-containing dual anti-HER2 therapy is superior to trastuzumab single agent for HER2-positive EBC independent of hormone receptor status. The correlation between survival and LN status needs further verification. Trastuzumab plus pertuzumab or plus neratinib is the preferred regimen with substantial efficacy and lower toxicity.

## 1. Introduction

Breast cancer is the most commonly diagnosed cancer and the leading cause of cancer-related deaths in women [1]. It is a heterogeneous disease and divided into four major molecular subtypes based on gene expression [2], of which the human epidermal growth factor receptor 2- (HER2-) positive subtype accounts for 15%–20% of breast cancers (BC) and is associated with a worse prognosis [3–5].

HER2 belongs to the human epidermal growth factor receptor (EGFR/HER/ErbB) family which also includes HER1 (EGFR), HER3, and HER4. HER receptors are transmembrane glycoproteins that comprise an extracellular ligand-binding region and an intracellular tyrosine kinase domain [6]. Trastuzumab (Herceptin), a monoclonal antibody against subdomain IV of the HER2extracellular domain (ECD), combined with chemotherapy can significantly improve the prognosis of HER2-positive BC patients compared with chemotherapy alone, which has been demonstrated in the Cochrane meta-analyses [7, 8]. Furthermore, according to several large and long-term follow-up trials, one year of trastuzumab therapy plus chemotherapy has become the standard of care for HER2-positive early breast cancer (EBC) patients [9–12]. However, cases of drug resistance remain and about 30% of patients relapse after trastuzumab therapy and new approaches are required [10–12].

Following trastuzumab, other HER2-targeting agents including lapatinib [13], pertuzumab [14], and neratinib [15] have been approved by the US Food and Drug Administration (FDA) for the treatment of HER2+ BC. Pertuzumab, another humanized monoclonal antibody, differs from trastuzumab in that it binds to the extracellular domain II of HER2 and inhibits homodimer or heterodimer formation, which has complementary mechanisms of action with trastuzumab to improve the efficacy of cancer therapy [16]. Lapatinib and neratinib are both oral, small molecule tyrosine kinase inhibitors that can further enhance HER2 inhibition by blocking intracellular signaling pathways [17]. The difference is that lapatinib is a dual reversible inhibitor of HER1 and HER2 tyrosine kinases, while neratinib is an irreversible inhibitor of HER1, HER2, and HER4.

Further studies focused on identifying biomarkers that may effectively predict which patients will respond best to HER2-targeted therapies. The I-SPY 2 trial, an adaptive phase 2 trial, identifying eight biomarker subtypes with considering HER2 status, hormone receptor status, and risk based on a 70-gene profile, found that neratinib was more likely to have an increased pathological complete response (pCR) rate than trastuzumab when added to standard chemotherapy in patients with HER2-positive (HER2+) and hormone-receptor-negative (HR−) BC [18]. Veeraraghavan et al. found that a clinical subtype in breast cancer with high HER2 amplification and an intact PI3K pathway has a better response to anti-HER2 therapies without chemotherapy [19]. The findings of Kim et al. showed that discordance between IHC-based subtypes and PAM50-based intrinsic subtypes was related to inadequate treatment and diminished survival in BC [20]. Studies also indicated that the percentage of stromal tumor-infiltrating lymphocytes (TILs) was associated with a higher pCR rate and improved survival in patients with HER2 + BC [21–23]. The optimal predictive biomarkers need further validation to contribute to development of precision medicine.

Clinical studies have shown that combining different anti-HER2 agents with complementary mechanisms may overcome drug resistance and be more effective than single-agent therapy. In the neoadjuvant setting, the NeoSphere trial confirmed dual blockade with trastuzumab plus pertuzumab produced a higher pathological complete response (pCR) which was pronounced in the hormone receptor-negative (HR−) patients [24]. The NeoALTTO trial demonstrated trastuzumab plus lapatinib therapy also significantly improved pCR [25]. In the adjuvant setting, the NCCN Guidelines recommended trastuzumab plus pertuzumab as an option for ≥T2 and ≥N1 HER2-positive patients because the APHINITY trial showed a substantial invasive disease-free survival (iDFS) benefit from trastuzumab plus pertuzumab, especially in lymph node-positive (LN+) patients [26, 27]. However, the ALTTO trial reported no substantial DFS benefit from trastuzumab plus lapatinib therapy and there was higher toxicity [28]. The use of dual anti-HER2 therapy and the most beneficial subgroups of patients as well as the correlated toxicities still needs further exploration.

Thus, we conducted a meta-analysis to evaluate the efficacy and safety of using trastuzumab-containing dual anti-HER2 regimens versus standard trastuzumab alone regimen in patients with HER2-positive EBC and to identify the optimal dual anti-HER2 regimens, as well as the subgroup of patients who would most likely benefit from dual therapy.

## 2. Methods

### 2.1. Eligibility Criteria

We included prospective phase II/III randomized controlled trials (RCTs) that assessed the efficacy or safety of trastuzumab-containing dual anti-HER2 therapy versus trastuzumab single-agent therapy in patients with HER2-positive EBC. We excluded patients with metastatic BC and studies with insufficient outcomes data.

### 2.2. Outcome Measures

The primary outcomes were event-free survival/invasive disease-free survival (EFS/iDFS) and overall survival (OS). The secondary outcomes were overall response rate (ORR), pCR rate in breast and axillary LNs, cardiac toxicity, and other toxicities. For definitions of outcomes, see Additional file 1: [Supplementary-material supplementary-material-1].

### 2.3. Search Strategy

We searched Cochrane Central Register of Controlled Trials (CENTRAL), EMBASE, MEDLINE, and ClinicalTrials.gov for eligible RCTs up to December 2018. We also screened relevant abstracts from the San Antonio Breast Cancer Symposium (SABCS), American Society of Clinical Oncology (ASCO), and European Society for Medical Oncology (ESMO) Meeting as well as related meta-analyses, reviews, and editorials of HER2-positive BC. The following keywords were adopted: breast cancer, trastuzumab (Herceptin), pertuzumab (Perjeta), lapatinib (Tykerb), neratinib (HKI-272), afatinib (BIBW-2992), and MM-111.

### 2.4. Data Extraction and Quality Assessment

Two authors extracted the data independently and assessed the quality of each trial according to the risk of bias tool of The Cochrane Collaboration [29] and any discrepancies were resolved by consensus or consulting a third author.

### 2.5. Data Synthesis

We estimated pooled hazard ratios (HRs) for survival outcomes (OS, EFS/iDFS) and risk ratios (RRs) for dichotomous outcomes (ORR, pCR, and toxicities) with 95% confidence intervals (CIs) using the inverse-variance method of RevMan5.3 software [29]. The random-effects model was adopted to combine heterogeneity across studies.

We used *χ*^2^ and *I*^2^ statistics to quantify heterogeneity. Significant heterogeneity existed if *p* < 0.10 or *I*^2^ > 50%. The following subgroup analyses were performed: treatment setting (neoadjuvant or adjuvant setting), dual anti-HER2 regimen (trastuzumab plus lapatinib, trastuzumab plus pertuzumab, or trastuzumab plus neratinib), chemotherapy regimen (taxane-containing non-anthracycline, anthracycline plus taxane, or others), LN status, and hormone receptor status. We carried out sensitivity analyses for main outcomes and those with substantial heterogeneity using the leave-one-out procedure. The impact of small-study and reporting bias was assessed using funnel plots and Begg's test through Stata/SE 11.2 software [30].

## 3. Results

### 3.1. Study Selection and Characteristics

**Figure 1:**
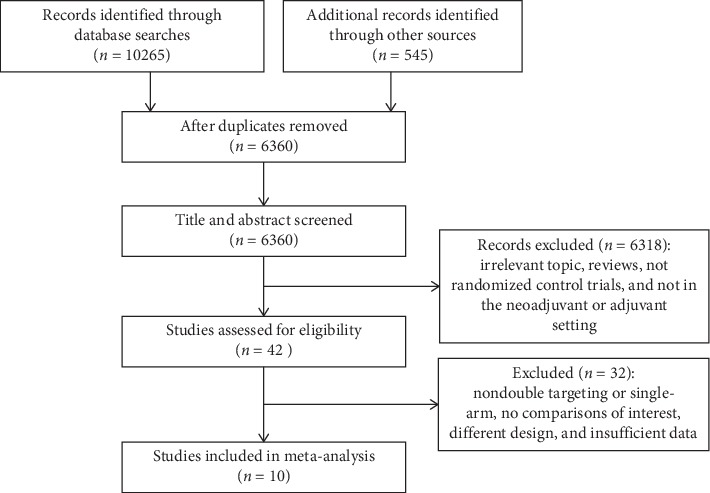
The process diagram of studies search and selection in the meta-analysis.

We searched and identified 10 studies corresponding to 16 publications with a total of 15,284 participants for the meta-analysis [24, 28, 31–42]. The flow diagram of study selection is shown in Figure 1. For characteristics of the included studies, see in Table 1. For details, see Additional file 1: [Supplementary-material supplementary-material-1].

The median follow-up time varied from 3.8 y to 6.9 y. Seven trials assessed the role of the dual HER2 blockade in a neoadjuvant setting [24, 31–33, 35, 36, 38], while three trials assessed the adjuvant setting [26, 28, 42]. There were seven trials of a trastuzumab plus lapatinib regimen [28, 31–33, 35, 36, 38], two trials considered a trastuzumab plus pertuzumab regimen [24, 26], and one trial considered a trastuzumab plus neratinib regimen [42]. Overall survival of the ExteNET trial was not reported [42]. The “risk of bias” assessment for each trial is shown in the Additional file 1: Appendix 3.

### 3.2. Effects of Interventions

The forest plots for all outcomes are included in [Supplementary-material supplementary-material-1] (Additional file 2).

#### 3.2.1. Overall Survival

**Figure 2:**
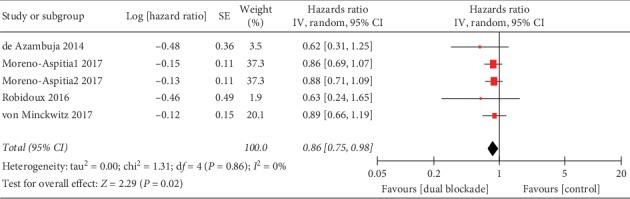
Overall survival of trastuzumab-containing dual anti-HER2 therapy, all studies. IV: inverse-variance method; random: random-effects model; Moreno-Aspitia1 2017: trastuzumab plus lapatinib group; Moreno-Aspitia2 2017: trastuzumab followed by lapatinib group.

Four studies reported data about OS for pooling in meta-analyses [26, 28, 31, 36], excluding that by Martin et al. that has not reached the planned 248 events [42]. The pooled OS data demonstrated a statistically significant improvement for patients who received trastuzumab-containing dual anti-HER2 therapy compared to trastuzumab single-agent therapy (HR 0.86, 95% CI 0.75–0.98, *p*=0.02; Figure 2). There was no heterogeneity across studies (*I*^2^ = 0%, *p*=0.86).

Subgroup analyses of treatment setting suggested that the survival benefit from the dual HER2 block was on the margins of statistical significance in adjuvant treatment (HR 0.87, 95% CI 0.65–1.00, *p*=0.05), but no significance in neoadjuvant treatment (HR 0.62, 95% CI 0.35–1.10, *p*=0.10). No subgroup differences were observed (interaction test, *p*=0.26). In a subgroup analysis according to type of dual HER2 blockade regimen, the dual therapy with trastuzumab plus lapatinib (HR 0.85, 95% CI 0.73–0.99, *p*=0.03) significantly improved the OS compared to trastuzumab plus pertuzumab (HR 0.89, 95% CI 0.66–1.19, *p*=0.42). However, there were no subgroup differences (interaction test, *p*=0.80).

#### 3.2.2. Event-Free Survival/Invasive Disease-Free Survival

**Figure 3:**
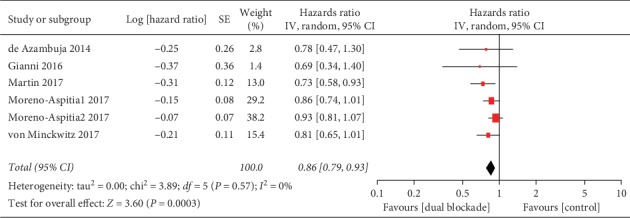
Event-free survival/invasive disease-free survival of trastuzumab-containing dual anti-HER2 therapy-all studies. IV: inverse-variance method; random: random-effects model; Moreno-Aspitia1 2017: trastuzumab plus lapatinib group; Moreno-Aspitia2 2017: trastuzumab followed by lapatinib group.

The EFS/iDFS was reported in 5/10 studies [24, 26, 28, 31, 42]. There was a substantial benefit with dual HER2 blocking (HR 0.86, 95% CI 0.79–0.93, *p*=0.0003; Figure 3) with no heterogeneity among studies (*I*^2^ = 0%, *p*=0.57).

**Figure 4:**
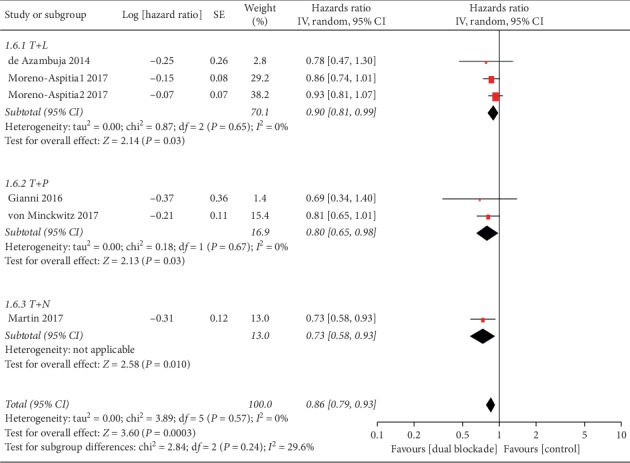
Event-free survival/invasive disease-free survival stratified by type of dual HER2 blockade regimen. T: trastuzumab; L: lapatinib; P: pertuzumab; N: neratinib; IV: inverse-variance method; random: random-effects model; Moreno-Aspitia1 2017: trastuzumab plus lapatinib group; Moreno-Aspitia2 2017: trastuzumab followed by lapatinib group.

Subgroup analyses of treatment setting indicated a substantial EFS/iDFS benefit with dual blockade in an adjuvant setting (HR 0.86, 95% CI 0.78–0.94, *p*=0.001) versus the neoadjuvant setting (HR 0.75, 95% CI 0.49–1.13, *p*=0.17), but no subgroup difference (interaction test, *p*=0.52). In a subgroup analysis according to type of dual anti-HER2 regimen, higher EFS/iDFS benefits were observed in the regimens with trastuzumab plus neratinib (HR 0.73, 95% CI 0.58–0.93, *p*=0.01; Figure 4) and trastuzumab plus pertuzumab (HR 0.80, 95% CI 0.65–0.98, *p*=0.03; Figure 4) than trastuzumab plus lapatinib (HR 0.90, 95% CI 0.81–0.99, *p*=0.03; Figure 4). However, no subgroup differences were found (interaction test, *p*=0.24).

**Figure 5:**
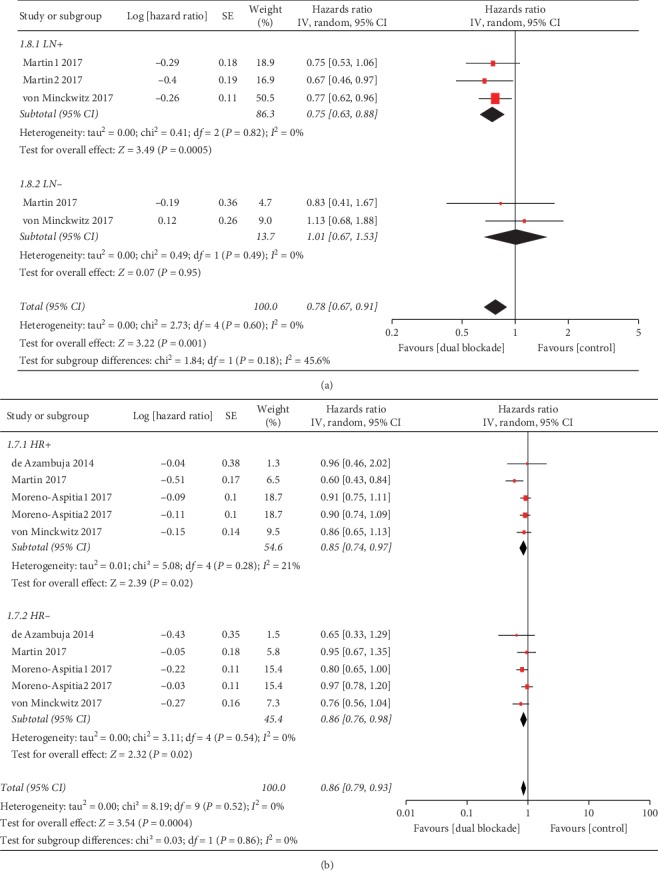
Subgroup analyses of event-free survival/invasive disease-free survival (EFS/iDFS). (a) EFS/iDFS stratified by lymph node status. LN+: lymph node positive; LN−: lymph node negative; Martin1 2017: subgroup of 1–3 positive LN; Martin2 2017: subgroup of ≥4 positive LN. (b) EFS/iDFS stratified by hormone receptor status. HR+: hormone receptor positive; HR−: hormone receptor negative; IV: inverse-variance method; random: random-effects model; Moreno-Aspitia1 2017: trastuzumab plus lapatinib group; Moreno-Aspitia2 2017: trastuzumab followed by lapatinib group.

Furthermore, we also found that the benefit of EFS/iDFS with a dual HER2 block in the LN + subgroup (HR 0.75, 95% CI 0.63–0.88, *p*=0.0005; Figure 5(a)) was superior to the LN-subgroup (HR 1.01, 95% CI 0.67–1.53, *p*=0.95; Figure 5(a)) but was not associated with the hormone receptor status (Figure 5(b)). However, the interaction test suggested that the EFS/iDFS benefit does not depend on LN status (*p*=0.18).

#### 3.2.3. Overall Response Rate

The ORR data from five studies were analyzed [24, 25, 35, 36, 38]. We excluded Guarneri et al. [33] in which the clinical objective response was reported as approximately 90% without further information. The difference in ORR did not reach statistical significance in either the pooled analysis (RR 1.03, 95% CI 0.96–1.10, *p*=0.45) or the subgroup analysis of the dual anti-HER2 regimen.

#### 3.2.4. Pathological Complete Response

Seven neoadjuvant studies reported pCR data [24, 31–33, 35, 36, 38]. The pCR rates for the dual-targeted group and monotherapy group were 51.60% and 38.26%. There was a significant 13.34% absolute improvement (RR 1.34, 95% CI 1.15–1.57, *p*=0.0002) with no substantial heterogeneity (*I*^2^ = 34%, *p*=0.17).

Subgroup analyses of dual anti-HER2 regimens showed a pCR rate favouring the regimen of trastuzumab plus pertuzumab (RR 1.83, 95% CI 1.19–2.81, *p*=0.006) versus trastuzumab plus lapatinib (RR 1.29, 95% CI 1.12–1.48, *p*=0.0003). A similar benefit was found in the HR− subgroup (RR 1.29, 95% CI 1.06–1.56, *p*=0.01) rather in the HR+ subgroup (RR 1.12, 95% CI 0.92–1.37, *p*=0.25) in the subgroup analysis of hormone receptor status. However, there were no subgroup differences between pCR and the type of dual anti-HER2 regimens or hormone receptor status (interaction test, *p*=0.13 and 0.34, resp.).

### 3.3. Safety

The forest plots for all outcomes are included in [Supplementary-material supplementary-material-1] (Additional file 2).

#### 3.3.1. Cardiac Toxicities

**Figure 6:**
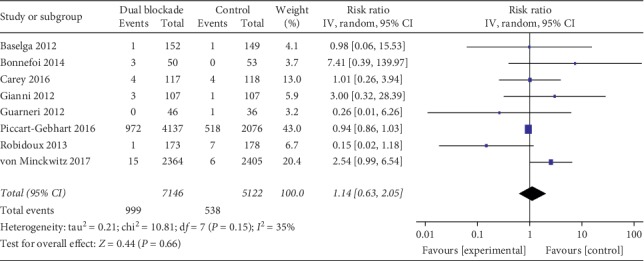
Cardiac toxicity of trastuzumab-containing dual anti-HER2 therapy-all studies. IV: inverse-variance method; random: random-effects model.

Eight studies assessing cardiotoxicity were pooled in the meta-analysis [24–26, 28, 32, 33, 36, 38]. There was no significant difference in cardiotoxicity between trastuzumab-containing dual-targeting therapy and trastuzumab alone therapy (RR 1.14, 95% CI 0.63–2.05, *p*=0.66, Figure 6).

In the subgroup analysis of a treatment setting, no significant cardiotoxicity was observed either in the neoadjuvant setting (RR 0.92, *p*=0.88) or in the adjuvant setting (RR 1.38, *p*=0.51). Subgroup analysis stratified by congestive heart failure (CHF) and left ventricular ejection fraction (LVEF) decline showed no substantial increase in CHF (RR 0.45, *p*=0.28) and LVEF decline (RR 0.95, *p*=0.31) in patients receiving dual-targeting therapy. Moreover, we performed subgroup analyses for CHF and LVEF, stratified by the type of dual anti-HER2 regimen and the type of chemotherapy, and no statistical difference was observed. In our meta-analysis, LVEF decline was defined as reported by the authors of included studies because different thresholds were used. More events in the APHINITY and ALTTO trials may be due to the fact that large enrolled population and broad definition of LVEF decline were used, so we also performed the corresponding analyses using the narrow definition of LVEF decline and the results also showed no significant statistical difference.

#### 3.3.2. Other Toxicities

We conducted analyses of other common grade 3/4 toxicities reported in more than half of the trials: diarrhea (10 studies), hepatic toxicity (9 studies), skin disorder (9 studies), neutropenia (8 studies), febrile neutropenia (7 studies), nausea and vomiting (5 studies), and fatigue (5 studies).

Patients receiving dual HER2 blocking therapy had a significant increase in the incidence of grade 3/4 diarrhea (RR 8.22, 95% CI 3.89–17.38, *p* < 0.00001), hepatic toxicity (RR 2.32, 95% CI 1.30–4.14, *p*=0.004), skin disorder (RR 4.20, 95% CI 2.40–7.34, *p* < 0.00001), and nausea and vomiting (RR 3.51, 95% CI 1.19–10.38, *p*=0.02). There were no statistical differences in the incidence of neutropenia, febrile neutropenia, or fatigue.

Subgroup analysis of dual anti-HER2 regimens and chemotherapy regimens was performed for each toxicity, the results showed that diarrhea was mainly associated with the trastuzumab plus neratinib group and trastuzumab plus lapatinib group, and hepatic toxicity and skin disorders were mainly associated with the trastuzumab plus lapatinib group, while nausea and vomiting were associated with the trastuzumab plus neratinib group. And a taxane-containing non-anthracycline regimen has a lower risk of diarrhea than an anthracycline plus taxane regimen. No other differences were observed in the subgroup analyses.

### 3.4. Sensitivity Analyses and Publication Bias

As most of the outcomes did not show significant heterogeneity, we carried out sensitivity analyses for OS, EFS/iDFS, ORR, pCR, and cardiac toxicity and the results were stable (Additional file 2: [Supplementary-material supplementary-material-1]). The funnel plots and Begg's test for OS and EFS/iDFS indicated no evidence of publication bias (Additional file 2: [Supplementary-material supplementary-material-1]).

## 4. Discussion

This meta-analysis of RCTs demonstrated that trastuzumab-containing dual anti-HER2 therapy was superior to standard trastuzumab alone therapy for HER2-positive EBC treatment, with a significant improvement in EFS/iDFS and OS.

Although dual anti-HER2 therapy has shown significant improvement in pCR in neoadjuvant treatment, our results demonstrated that the benefit of dual-targeting therapy in the neoadjuvant treatment did not extend to the long-term survival benefits, a significant DFS and OS benefit in favour of the adjuvant treatment versus the neoadjuvant treatment. Despite no substantial heterogeneity was found in all pooled analyses, differences between studies might be relevant. Firstly, differences in duration of dual-targeted treatment are as follows: all three studies included in the adjuvant setting have completed a 1-year dual anti-HER2 therapy [26, 28, 42], while there was only one of seven studies in the neoadjuvant setting [31]. Secondly, differences in included populations are as follows: in the adjuvant treatment, the population recruited in the APINITY trial and the ExteNET trial were relatively high-risk (with more LN+ patients, 63% and 77%, resp.), which were more likely to report positive results in the adjuvant setting. However, no interaction between survival and treatment setting was observed (interaction test for EFS/iDFS and OS, *p*=0.52 and 0.26). Thus, some caution is still required.

When taking hormone receptor status into consideration, several meta-analyses of neoadjuvant treatment demonstrated that the pCR rate was significantly improved in patients receiving dual HER2 block versus trastuzumab alone and higher in HR patients [43–46]. In adjuvant therapy, the APINITY and ALTTO trials suggested that the dual-targeted therapy could significantly enhance EFS/iDFS in HR patients [26, 28]. These results of previous studies seem to indicate that HR patients can benefit more from dual anti-HER2 therapy. Nonetheless, subgroup analysis of hormone receptor status in our meta-analysis found no difference in EFS/iDFS between the two groups. Even if the pCR was more pronounced in HR patients, no interaction was found (interaction test, *p*=0.34). Therefore, hormone receptor status may not be a determinant of a dual-targeted selective therapy. Or as described in the CALGB 40601 study, we should pay more attention to the subtype than hormone receptor status when predicting pCR [32]. Of note, the ExteNET trial suggested that neratinib administered after trastuzumab significantly improved iDFS in hormone receptor-positive (HR+) patients with HER2-positive BC. This may be a consequence of there being no cross-resistance for neratinib and trastuzumab in the HR + patients, or the interaction of neratinib with hormones reversed the upregulation of estrogen receptors caused by trastuzumab to modify HER2 resistance [42]. With results diametrically opposite to other studies, we conducted an extra subgroup analysis excluding the ExteNET study and the results did not change.

The LN status, another important factor affecting the clinical treatment decisions, has been shown in clinical studies that LN + patients are more likely to benefit from dual-targeting therapy [24, 42], but our results suggested that, despite the more pronounced EFS/iDFS benefit in LN + patients, there was no significant interaction between survival and LN status (*p*=0.18). Similarly, a recent meta-analysis assessing the optimal duration of trastuzumab treatment also showed no significant interaction between survival and HR status or LN status (*p* for interaction test, 0.26 and 0.60) [47]. The guidelines recommend using an interaction test for subgroup analyses, as evidenced that inappropriate subgroup-specific analysis was of low reliability and the problem may be underestimated [48]. Thus, the subgroup results should be interpreted carefully.

In addition to the above, different combination regimens of dual HER2 block might affect efficacy. Subgroup analysis of the type of dual anti-HER2 regimen revealed that OS was significantly improved with trastuzumab plus lapatinib, while the effect on EFS/iDFS did not differ significantly among the three groups. Although the OS benefit with trastuzumab plus lapatinib might be somewhat unexpected considering the negative results of the ALTTO trial, the following points in the ALTTO trial should be noted, except for the unreported final OS results of the ExteNET trial: First, the recruited patients were designed for DFS, with a low risk of recurrence (more LN− (40%) and HR+ (57%) patients than the other included trials), which may explain the lower-than-expected DFS event [40]. Second, a time-driven analysis was conducted to obtain early results rather than a more mature event-driven analysis [49]. Third, due to the toxicity of lapatinib, the lapatinib group was closed early and the proportion of patients who completed the planned dose in the dual-targeting group was lower. Finally, studies demonstrated that intermittent administration of lapatinib is more effective than continuous administration [50, 51]. All of above may affect statistical power and result in negative results [49, 52]. Notably, in the ALTTO trial, a protocol modification required *p* ≤ 0.25 because of the early closure of the lapatinib group, while we considered *p* ≤ 0.05 to be statistically significant [28]. And it is statistically possible that the pooled analysis showed a marginally significant result after expanding the sample size by integrating several trials that are close to meaningful. Additionally, the meta-analysis by Debiasi et al. [53] also found that chemotherapy plus trastuzumab plus lapatinib was probably the first choice for improving OS compared to chemotherapy plus trastuzumab with a posterior probability of 62.47%. Trastuzumab plus neratinib was the best strategy for DFS, with a posterior probability of 50.55%. These results coincided with ours, but our meta-analysis also included the mature OS results of the APINITY trial. It seems that there might be differences among the three dual anti-HER2 regimens in terms of EFS/iDFS and OS, but no significant interactions were observed (*p*=0.24 and 0.80, resp.). More RCTs are needed to confirm the best combination regimen due to the limited number of trials included in each subgroup.

Regarding the toxicities, the risk of cardiac toxicity did not increase, as described in other meta-analyses [43, 45, 46], which increases our confidence in using dual-targeted therapy. However, the incidence of grade 3/4 diarrhea, hepatic toxicity, nausea and vomiting, and skin disorders was significantly increased. Subgroup analysis of dual anti-HER2 regimen showed that the toxicities in the lapatinib group were mainly diarrhea, hepatic toxicity, and skin disorders, and the main toxicities for the neratinib group were diarrhea, nausea and vomiting, and fatigue, while for the pertuzumab group the main toxicity was diarrhea. Almost all trials that contained treatment with lapatinib reported a dose reduction, termination of treatment, and even early closure of the treatment group due to the high risk of adverse events (AEs) that can also be seen in other published meta-analyses [45, 54, 55]. Conversely, most of the cases of diarrhea reported in the neratinib-containing group were of low grade and were preventable and tolerable despite the high incidence. The risk of AEs in the pertuzumab-containing group was significantly lower than that in the lapatinib group and the neratinib group. Therefore, we believe that trastuzumab plus lapatinib would be the most effective regimen if the patients could tolerant the toxicity. If they cannot, then trastuzumab plus pertuzumab or plus neratinib would be the preferred options for HER2-positive EBC after weighing the effects and safety. We are still waiting for the final OS result of the ExteNET trial and more trials using dual HER2 blocking with trastuzumab plus pertuzumab or plus neratinib.

Our manuscripts collected comprehensive and latest clinical data to make up for the deficiencies of previous studies and present the most cutting-edge results in this field. We compared dual anti-HER2 therapy with the current standard care (trastuzumab alone) for treating HER2-positive EBC and comprehensively evaluated efficacy and safety, the neoadjuvant and adjuvant setting, and corresponding subgroup analyses to look for the populations that would most benefit to identify crucial personalize therapy.

Nevertheless, shortcomings remain. Firstly, the heterogeneous nature of the patients, the clinical settings, and the drugs in this meta-analysis may reduce reliability. However, we conducted the pooled analyses, several correlation subgroup analyses, and sensitivity analyses, and the results did not show any significant heterogeneity. Secondly, for data available for the regimen with trastuzumab plus neratinib or plus pertuzumab, hormone receptor status and LN status were limited. And neratinib was administered after completion of trastuzumab-based adjuvant therapy rather than being used simultaneously in the ExteNET trial; further RCTs are still needed to focus on trastuzumab plus pertuzumab or plus neratinib regimens and LN status and hormone receptor status to improve our understanding. Finally, EFS/iDFS and OS can be affected by subsequent adjuvant therapy such as the regimens and duration of treatment.

## 5. Conclusions

We conclude that the trastuzumab-containing dual HER2 block is superior to standard trastuzumab alone for patients with HER2-positive EBC. Although the dual HER2 block was associated with a higher risk of grade 3/4 AEs, especially in the lapatinib group, there was no increase in cardiotoxicity. Trastuzumab combined with lapatinib achieved the greatest OS benefit but is accompanied by higher AEs. Weighing the pros and cons, trastuzumab plus pertuzumab or plus neratinib is the preferred choice with substantial benefit and lower toxicity, a result still waiting for the final OS results of the ExteNET trial. Notably, the survival was independent of hormone receptor status, and the correlation between survival and LN status should be interpreted cautiously. Further investigations are needed to determine the best dual anti-HER2 regimen and the subgroup populations that will benefit most.

## Figures and Tables

**Table 1:** Characteristics of the included studies.

				Neoadjuvant treatment		Adjuvant treatment						
Study	Phase	*N*	MF	Chemotherapy (wks)	Anti-HER2 therapy	Chemotherapy (wks)	Anti-HER2 therapy	Arms	LN+ patients (%)	HR+ patients (%)	Duration^#^ (wks)	Outcomes
*Adjuvant setting*													
ExteNET^*∗*^	III	2840	5.2 y	UNK	T or none	UNK	T + N	1420	1085 (76)	816 (57)	52	DFS, IDFS, OS, and safety
(Martin1 2017^a^; Martin2 2017^a^; Chan 2016)							T	1420	1084 (76)	815 (57)		
ALTTO^*∗*^	III	8381	6.9 y	UNK	None	Design 1: chemotherapy × (12–18) Design 2: A × (9–12) + Taxane × 12 Design 2B: (Doc + Carb) × 18	T + L^b^	2093	1080 (52)	1203 (57)	52	DFS, OS, and safety
(Piccart-Gebhart 2016; Moreno-Aspitia1 2017^b^; Moreno-Aspitia2 2017^b^)							T⟶L^b^	2091	1078 (52)	1205 (58)	T12⟶L34	
							T	2097	1072 (51)	1200 (57)	52	

APHINITY (Von Minckwitz 2017)	III	4805	3.8 y	None	None	FEC × (9–12) + Doc/Pal × 12; or AC/EC × (8–12) + Doc/Pal × 12; or (Doc + Carb)×18	T + P	2400	1503 (63)	1536 (64)	52	IDFS, DFS, OS, and safety
							T	2405	1502 (62)	1546 (64)		

*Neoadjuvant setting*												
EORTC 10054	IIb	128	NR	Doc × 9 + FEC × 9	L + T	NR	T	52	33 (64)	25 (48)	9	pCR (breast + nodes), pCR (breast), response rates, and safety
(Bonnefoi 2014)					T			53	36 (68)	27 (51)		

CALGB 40601	III	295	NR	wP × 16	L + T	AC × (8–12)	T	117	NR	69 (59)	16	pCR (breast + nodes) and safety
(Carey 2016)					T			118		70 (59)		

NeoALTTO^*∗*^	III	455	3.84 y	wP × 12w	L + T	FEC × 9	T + L	152	UNK	77 (51)	52	pCR (breast + nodes), pCR (breast), ORR, safety, DFS, EFS, and OS
(Baselga 2012; de Azambuja 2014)					T		T	149		75 (50)		

CHER-LOB^*∗*^	IIb	121	NR	wP × 12 + FEC × 12	L + T	NR	T	46	NR	28 (61)	26	pCR (breast + nodes) and clinical objective responses
(Guarneri 2012; Guarneri 2015)					T			36		21 (58)		

NeoSphere^*∗*^	II	417	5 y	D × 12	T + P	FEC × 9	T	107	75 (70)	50 (47)	12	pCR(breast) and pCR (breast + nodes)
(Gianni 2012; Gianni 2016)				None	T + P	D × 12 <+ FEC × 9		107	75 (70)	51 (48)		Clinical response rate, safety, PFS, and DFS
				D × 12	T	FEC×9		107	75 (70)	50 (47)		

LPT109096	II	100	NR	FEC × 12 + wP × 12	T + L	UNK	UNK	33	20 (61)	20 (61)	26	pCR (breast + nodes), clinical complete response (CCR), and safety
(Holmes 2013)					T			33	15 (45)	15 (45)		

NSABP B-41^*∗*^	III	519	5 y	AC × 12 + wP × 12	T + L	None	T	174	85 (49)	108 (62)	12	pCR(breast), pCR (breast + nodes), clinical complete response, safety, and OS
(Robidoux 2013; Robidoux 2016)					T			181	92 (51)	122 (67)		

MF: median follow-up; A: anthracycline; FEC: fluorouracil + epirubicin + cyclophosphamide; Doc: docetaxel; Carb: carboplatin; Pal: paclitaxel; wP: weekly paclitaxel; wk: week; HR+: hormone receptor positive; LN+: lymph node positive; T: trastuzumab; L: lapatinib; P: pertuzumab; N: neratinib; T + L: trastuzumab plus lapatinib; T ⟶ L: trastuzumab followed by lapatinib; NR: unreported; UNK: unknown. ^#^Duration of dual anti-HER2 therapy.^*∗*^Studies with more than one publication. ^a^In the ExteNET trial (Martin 2017), lymph node- (LN-) positive patients were divided into two subgroups, 1–3 positive LN (Martin1 2017) and ≥4 positive LN (Martin2 2017), for the subgroup analysis of LN status. ^b^ In the ALTTO trial (Moreno-Aspitia 2017), there were two experimental groups (trastuzumab plus lapatinib group (Moreno-Aspitia1 2017) and trastuzumab followed by lapatinib group (Moreno-Aspitia2 2017).

## Data Availability

The data used to support the findings of this study are included within the supplementary information files.

## Supplementary Materials

Supplementary Materials Additional file 1: Appendix 1: definitions of outcomes. Appendix 2: characteristics of included studies. Appendix 3: risk of bias assessment. Additional file 2: Figure S1: forest plots for all outcomes. Figure S2: sensitivity analyses. Figure S3: publication bias.
