# Supplementary material for: Dual HER2 Blockade versus a Single Agent in Trastuzumab-Containing Regimens for HER2-Positive Early Breast Cancer: A Systematic Review and Meta-Analysis of Randomized Controlled Trials
Source: J Oncol. 2020 Mar 16;2020:5169278. doi: 10.1155/2020/5169278 (PMC7102417; doi:10.1155/2020/5169278)
Supplement: Supplementary Materials — Additional file 1: Appendix 1: definitions of outcomes. Appendix 2: characteristics of included studies. Appendix 3: risk of bias assessment. Additional file 2: Figure S1: forest plots for all outcomes. Figure S2: sensitivity analyses. Figure S3: publication bias. [file 5169278.f1.zip › 5169278.f1/Additional file 1.doc]

**Supplementary material (Additional file 1)**

**目录**

Appendix 1. Definitions of outcomes	2

Appendix 2. Characteristics of included studies [ordered by study ID]	3

Appendix 3. Risk of bias assessment	16

# Appendix 1. Definitions of outcomes

**1. Overall survival (OS):** time from randomization to death from any cause.

**2. Event-free survival (EFS):** time from randomization to the date of breast cancer relapse after surgery, second primary cancer, or death from any cause.

**3. Invasive disease-free survival (iDFS):** time from randomization until the date of the first occurrence of the following disease-free survival events: invasive ipsilateral breast tumor recurrence, invasive contralateral breast cancer, local/regional invasive recurrence, distant recurrence and death from any cause.

**4.** **Overall response rate (ORR):** confirmed complete response (CR) and partial response (PR). ORR was assessed based on the World Health Organization (WHO) criteria or the Response Evaluation Criteria in Solid Tumors (RECIST) version 1.0.

**5. Pathologic complete response (pCR)** in breast and ipsilateral axillary lymph nodes: no invasive neoplastic cells at microscopic examination of the primary tumor and axillary lymph nodes at surgery (ypT0/is ypN0). Remaining in-situ lesions were allowed.

**6. Cardiac toxicity:** congestive heart failure (CHF) and decline of left ventricular ejection fraction (LVEF). CHF defined as Cardiac dysfunction New York Heart Association Class III or IV events; severe CHF; symptomatic CHF; or confirmed CHF. LVEF decline was defined as reported by the authors of included studies because of different thresholds were used.

**7. Other toxicities:** neutropenia (grade 3/4), febrile neutropenia (grade 3/4), diarrhea (grade 3/4), hepatic toxicity (grade 3/4), Skin disorder (grade 3/4), nausea and vomiting (grade 3/4), and fatigue (grade 3/4). These were defined and graded according to the World Health Organization (WHO) and National Cancer Institute (NCI) criteria.

# Appendix 2. Characteristics of included studies [ordered by study ID]

**De Azambuja 2014**

| Methods | Accrual time: Jan 2008 to May 2010  Parallel groups, randomised, open-label, multicentre, phase 3 trial  Baseline comparability: balanced |
| --- | --- |
| Participants | 455 female enrolled  Median age: Trastuzumab and lapatinib arm: 50 (43-59), Trastuzumab arm: 49 (44-57)  Diagnosis: HER2-positive primary breast cancer with tumors greater than 2 cm in diameter  Inclusion criteria: Histologically confirmed invasive breast cancer with HER2 overexpression or amplification as per guidelines. HER2 status was assessed locally at participating institutions that had been accredited by our certified laboratory (Vall d Hebron Institute of Oncology). Participating patients had primary breast tumors greater than 2 cm in diameter measured by either mammography or echography. Patients had to have adequate baseline hepatic, renal, cardiac, and bone marrow function for inclusion. Adequate cardiac function was defined as a baseline left ventricular ejection fraction of 50% or more measured by echocardiography or multiple gates  HER2+: 100% |
| Interventions | 3-arm RCT  Trastuzumab and lapatinib arm (randomised N = 152): Oral lapatinib 1000 mg daily plus trastuzumab 4 mg/kg IV load followed by 2 mg/kg IV weekly for 6 weeks, followed by lapatinib 750 mg daily plus trastuzumab 2 mg/kg IV weekly plus weekly paclitaxel 80 mg/m^2 IV for an additional 12 weeks  Trastuzumab arm (randomized N = 149): Trastuzumab 4 mg/kilograms [kg] IV load followed by 2 mg/kg IV weekly for 6 weeks, followed by trastuzumab plus weekly paclitaxel (80 mg/m^2 IV) for an additional 12 weeks  Lapatinib arm (randomized N = 154): Oral lapatinib (1500 milligrams [mg] daily) for 6 weeks, followed by lapatinib plus weekly paclitaxel (80 mg per meters squared [mg/m^2]) intravenously (IV) for an additional 12 weeks |
| Outcomes | Primary: pCR (in the breast)  Secondary: locoregional total pCR (in the breast and axillary lymph nodes), objective tumor response rate (complete response and/or partial response were evaluated using World Health Organization (WHO) criteria by clinical examination and by mammography and breast echography with bi-dimensional measurements at Week 6), patients with node-negative disease at surgery, patients having breast-conserving surgery; rate of conversion to breast-conserving surgery, safety and tolerability, event-free survival (EFS), OS (overall survival), the association between pathological complete response and event-free or overall survival, molecular characteristics of responding tumours, and biomarker expression. |
| Notes | Study ID: NeoALTTO  Other publication: Baselga 2012  ClinicalTrials identifier: NCT00553358   - Median survival follow-up: 3.84 years - Protocol amendment 3 also replaced disease-free survival with event-free survival to align with recommendations from the FDA. - To reduce the occurrence of diarrhea, the lapatinib dose was reduced from 1000 mg to 750 mg when paclitaxel was added to the combination (protocol amendment 2, approved Oct 10, 2008). - 12 (8.1%) in trastuzumab group and 60 (39.5%) lapatinib plus trastuzumab group not completed as planned. - Patients were analyzed according to intention to treat analysis for pCR in the breast, EFS, OS and objective tumor response rate.145/152 women received trastuzumab plus lapatinib, 145/149 women received trastuzumab alone for locoregional total pCR. 149/152 women received trastuzumab plus lapatinib, 148/149 women received trastuzumab alone for safety. |

**Bonnefoi 2014**

| Methods | Accrual time: October 2010 to January 2013  Randomized multicenter phase II b study  Baseline comparability: balanced |
| --- | --- |
| Participants | 128 female enrolled  Median age: Trastuzumab and lapatinib arm: 49.4(27.3-70.8), Trastuzumab arm: 47 (25.3-68.9)  Diagnosis: locally advanced, inflammatory or large operable HER2-positive primary breast cancer  Inclusion criteria: age 18 to 70 years, any large operable cT2 or cT3 or locally advanced or inflammatory breast cancer (clinical T4 a, b, c, d, any N or any T, N2 or N3), M0, histologically confirmed diagnosis of invasive breast cancer (patients with bilateral breast cancer were eligible if only one side was HER2-positive), HER2 positive tumor (immunohistochemistry 3+, or IHC 2+ and FISH/CISH +, or FISH or CISH+ only), availability of three tumour biopsies (one fixed in formalin and two frozen) before inclusion, WHO performance status of 0 to 2, adequate baseline cardiac evaluation (including left ventricular ejection fraction(LVEF)measured by echocardiography or multiple gate acquisition scan within the institutional range of normal), hepatic, renal, and bone marrow function  HER2+: 100% |
| Interventions | 3-arm RCT  Trastuzumab + lapatinib + docetaxel arm (randomized N = 52): 3 cycles of docetaxel (100 mg/m²) + trastuzumab weekly schedule+lapatinib (1000 mg/d) followed by 3 cycles of FEC 100  Trastuzumab + docetaxel arm (randomized N = 53): 3 cycles of docetaxel (100 mg/m²) + trastuzumab weekly schedule followed by 3 cycles of FEC 100  Lapatinib + docetaxel arm (randomized N = 23): 3 cycles of docetaxel (100 mg/m²) + lapatinib (1000 mg/d) followed by 3 cycles of FEC |
| Outcomes | Primary: pCR rate (ypT0/is)  Secondary: pCR rate (ypT0/is ypN0), clinical response rates, rate of breast conserving surgery, safety and toxicity |
| Notes | Study ID: EORTC 10054  ClinicalTrials identifier: NCT00450892  Median follow-up: not specified   - In June 2012, accrual in the lapatinib arm was closed for futility after the publication of several reports suggesting that lapatinib monotherapy plus chemotherapy was either less effective or not inferior to but more toxic than chemotherapy plus trastuzumab. - Six patients were excluded from the efficacy population: five because of a major violation of an eligibility criterion (a metastatic disease in one, over 70 years old in one, HER2-negative tumor after reassessment at the treating center in three), two of which did not start the allocated treatment. Additionally, one patient refused to start the allocated treatment. |

**Carey 2016**

| Methods | Accrual time: December 2008 to February 2012  Randomized Phase 3 Trial  Baseline comparability: not reported |
| --- | --- |
| Participants | 305 patients (female and male) enrolled  Median age: Trastuzumab and lapatinib arm: 48 (24-70), Trastuzumab arm: 50 (30-75)  Diagnosis: newly diagnosed, non-inflammatory, histologically confirmed, untreated clinical stage II to III HER2-positive disease  Inclusion criteria: Patients were age ≥ 18 years, had tumors ≥ 1 cm in size and had a pretreatment left ventricular ejection fraction ≥ 50%. Patients with multicentric or bilateral disease were eligible if the target lesion met other eligibility criteria.  HER2+: 100% |
| Interventions | 3-arm RCT  Trastuzumab + lapatinib arm (randomized N = 118): trastuzumab 2 mg/kg IV and paclitaxel 80 mg/m^2 IV over 1 hour once weekly and lapatinib ditosylate 750 mg PO once daily for 16 weeks  Trastuzumab arm (randomized N = 120): trastuzumab 2 mg/kg IV and paclitaxel 80 mg/m^2 IV over 1 hour once weekly for 16 weeks  Lapatinib arm (randomized N =67): lapatinib ditosylate 15000 mg PO once daily and paclitaxel 80 mg/m^2 IV over 1 hour once weekly for 16 weeks |
| Outcomes | Primary: pCR (in the breast)  Secondary: pCR (in breast and ipsilateral axillary lymph nodes), adverse events, OS (overall survival), iDFS (invasive disease-free survival was defined as the time from surgery until local or distant recurrence, new primary, or death from any cause, whichever was first.) |
| Notes | Study ID: CALGB 40601  Other publications: Krop 2018  ClinicalTrials identifier: NCT00770809  Median iDFS follow-up: 4.6 years   - Because of emerging data from several trials regarding excessive diarrhea, lapatinib was reduced from 1,000 to 750 mg in the THL arm in April 2010, after 34 patients were accrued. - In July 2011, based on reports of inferiority and greater toxicity of lapatinib-only regimens, the Lapatinib plus paclitaxel arm was closed. - There was 1 patient in each group withdrew consent prior to surgery and patients who did not undergo surgery were considered non-pCR. |

**Gianni 2016**

| Methods | Accrual time: December 2007 to December 2009  Randomized, multicentre, international, open-label, phase 2 study  Baseline comparability: balanced |
| --- | --- |
| Participants | 417 female enrolled  Median age: Trastuzumab and pertuzumab arm: 50 (28-77), Trastuzumab arm: 50 (32-74)  Diagnosis: women with locally advanced, inflammatory, or early HER2-positive breast cancer (no prior chemotherapy)  Inclusion criteria: All eligible patients had centrally confirmed HER2-positive, operable (T2-3, N0-1, M0), locally advanced (T2-3, N2-3, M0 or T4a-c, any N, M0), or inflammatory (T4d, any N, M0) breast cancer with primary tumors larger than 2 cm in diameter, were aged 18 years or older, and had not received any previous cancer therapy. Tumors had to be HER2 immunohistochemistry 3+ or 2+ and positive for fluorescence or chromogenic in-situ hybridization. Other main inclusion criteria were: baseline Eastern Cooperative Oncology Group (ECOG) performance status of 0 or 1, baseline left ventricular ejection fraction (LVEF) of 55% or more, as measured by echocardiography or multiple gated acquisition (MUGA).  HER2+: 100% |
| Interventions | 4-arm RCT  Trastuzumab and pertuzumab + docetaxel arm (randomized N = 107): trastuzumab IV infusion at a loading dose of 8 mg/kg and pertuzumab IV infusion at a loading dose of 840 mg on Day 1 of Cycle 1 (21-day cycle) followed by a maintenance dose of 6 mg/kg trastuzumab and 420 mg pertuzumab on Day 1 of Cycles 2-4. Docetaxel IV infusion was administered at a starting dose of 75 mg/m^2 on Day 1 of Cycle 1 followed by 100 mg/m^2 on Day 1 of Cycles 2-4, if no dose limiting toxicity occurred.  Trastuzumab and pertuzumab arm (randomized N = 108): trastuzumab IV infusion at a loading dose of 8 mg/kg and pertuzumab IV infusion at a loading dose of 840 mg on Day 1 of Cycle 1 (21-day cycle) followed by a maintenance dose of 6 mg/kg trastuzumab and 420 mg pertuzumab on Day 1 of Cycles 2-4.  Trastuzumab + docetaxel arm (randomized N = 107): trastuzumab intravenous (IV) infusion at a loading dose of 8 milligrams per kilogram (mg/kg) on Day 1 of Cycle 1 (21-day cycle) followed by a maintenance dose of 6 mg/kg on Day 1 of Cycles 2-4 and docetaxel IV infusion at a starting dose of 75 milligrams per square meter (mg/m^2) on Day 1 of Cycle 1 followed by 100 mg/m^2 on Day 1 of Cycles 2-4, if no dose limiting toxicity occurred.  Pertuzumab + docetaxel arm (randomized N = 94): pertuzumab IV at a loading dose of 840 mg on Day 1 of Cycle 1 (21-day cycle) followed by a maintenance dose of 420 mg on Day 1 of Cycles 2-4. Docetaxel IV infusion was administered at a starting dose of 75 mg/m^2 on Day 1 of Cycle 1 followed by 100 mg/m^2 on Day 1 of Cycles 2-4, if no dose limiting toxicity occurred |
| Outcomes | Primary: pCR in the breast  Secondary: clinical response rate (confirmed CR + PR), time to clinical response, breast-conserving surgery rate, safety, progression-free survival (defined as the time from the date of randomization to the first documentation of progressive disease or death; equivalent to event-free survival), disease-free survival (time from the first date of no disease [ie, date of surgery] to the first documentation of progressive disease or death), and the association between total pathological complete response (in the breast and axillary nodes) and progression-free survival |
| Notes | Study ID: NeoSphere  Other publications: Gianni 2012  ClinicalTrials identifier: NCT00545688  Median follow-up: trastuzumab arm: 60.5months(53-62), pertuzumab and trastuzumab arm: 61.2 months (59-63)   - One patient randomly assigned to group D received group A treatment, one patient assigned to group D received group B treatment, and one patient assigned to group B received group C treatment in error. - The rate of discontinuation in trastuzumab arm and trastuzumab plus lapatinib arm was 8.1% and 39.5%, respectively. - 392 (94%) of 417 patients had surgery as planned, and all those who did so had a valid assessment of pathological response. - Patients were analyzed according to intention to treat analysis for PCR, PFS and safety. Patients analyzed for DFS: pertuzumab and trastuzumab arm (randomized N=101), trastuzumab arm (randomized N=103). Patients analyzed for overall response (in all breast tumors and nodes examined): pertuzumab and trastuzumab arm (randomized N=100), trastuzumab arm (randomized N=97) |

**Guarneri 2012**

| Methods | Accrual time: August 2006 to November 2010  Prospective, randomized, multicenter, phase IIb  Baseline comparability: balanced |
| --- | --- |
| Participants | 121 female enrolled  Median age: Trastuzumab and lapatinib arm: 49(26-65), Trastuzumab arm: 50(34-65)  Diagnosis: HER2-positive, stage II to IIIA operable breast cancer  Inclusion criteria: previously untreated, infiltrating primary breast cancer of more than 2.0 cm in largest clinical diameter, HER2 positivity (either immunohistochemistry 3+or fluorescent in situ hybridization amplification), age 18 to 65 years, Eastern Cooperative Oncology Group performance status of 0 to 1, availability of tumor tissue for biologic and molecular examination before starting primary treatment, left ventricular ejection fraction (LVEF) within the institutional range of normal, normal organ and marrow function (leukocytes ≥3,000/uL, absolute neutrophil count ≥1,500/uL, platelets ≥100,000/uL, total bilirubin ≤1.5 times the upper limit of normal, and AST and ALT ≤2.5 times the upper limit of normal), ability to swallow and retain oral medication, adequate contraception methods for women of childbearing potential.  HER2+: 100% |
| Interventions | 3-arm RCT  Trastuzumab and lapatinib arm (randomized N = 46): trastuzumab 4 mg/kg IV on the day of the first paclitaxel course followed by 2 mg weekly and lapatinib 1000 mg/day orally for 26 weeks prior to surgery  Trastuzumab arm (randomized N = 36): trastuzumab 4mg loading dose followed by 2 mg weekly for 26 weeks prior to surgery  Lapatinib arm (randomized N = 39): lapatinib 1500 mg/day orally for 26 weeks prior to surgery  All participants received chemotherapy, which included paclitaxel 80 mg/m^2 weekly for 12 weeks, followed by IV fluorouracil 600 mg/m^2, IV epidoxorubicin 75 mg/m^2, and IV cyclophosphamide 600 mg/m^2, once every 21 days for four treatment courses. Trastuzumab or lapatinib was administered throughout the course of the CT and for two weeks after the last CT administration. |
| Outcomes | Primary: pCR (in breast and axillary nodes)  Secondary: clinical objective responses (CR and PR) in the breast, breast-conserving surgery rate, safety, the time to treatment failure, the percentage of inhibition of intermediate and final biomarkers of the proliferative and apoptosis pathways induced by the different combinations, and the correlation between gene expression at diagnosis and pathologic response. |
| Notes | Study ID: CHER-LOB  Other publications: Guarneri 2015  ClinicalTrials identifier: NCT00429299  Median follow-up: not specified   - After the second safety report of the independent data monitoring committee, the protocol was amended to reduce lapatinib doses to 1,250 mg PO daily in lapatinib arm and to 750 mg daily in trastuzumab and lapatinib arm because of the occurrence of grade 3 diarrhea in 20% of patients in lapatinib arm and in 41% of the patients in trastuzumab and lapatinib arm. - Twelve patients in lapatinib arm and eight patients in trastuzumab and lapatinib arm permanently discontinued lapatinib arm due to toxicity. - Two patients were excluded from efficacy analysis because of informed consent withdrawal (one patient in lapatinib arm) and major protocol violation (one patient in trastuzumab and lapatinib arm). - The safety analyses included all randomly assigned patients. Patients analyzed for pCR: trastuzumab and lapatinib arm (randomized N=45), trastuzumab arm (randomized N=36). Patients analyzed for clinical objective responses: not reported. |

**Holmes 2013**

| Methods | Accrual time: August 2007 to October 2010  Open-label, randomized, phase II study  Baseline comparability: all characteristics equal with the exception of hormone-receptor status. There were fewer HR-negative patients in the combination arm than in trastuzumab group (respectively: 39% and 55%) |
| --- | --- |
| Participants | 100 female enrolled  Median age: Trastuzumab and lapatinib arm: 50 (28-66), Trastuzumab arm: 54 (21-67)  Diagnosis: HER2-positive stage II or III invasive breast cancer  Inclusion criteria: ≥18 years of age; the presence of untreated, biopsy-proven, HER2-positive (immunohistochemistry [IHC] 3+ or fluorescence in situ hybridization [FISH] ratio >2.2), American Joint Commission on Cancer stage II or III invasive breast cancer; Eastern Cooperative Oncology Group (ECOG) performance status of 0 or 1; left ventricular ejection fraction ≥50% and within the institutional range of normal by echocardiogram or multi-gated acquisition scan; the ability to swallow and retain oral medication.  HER2+: 100% |
| Interventions | 3-arm RCT  Trastuzumab + lapatinib arm (randomised N = 33): trastuzumab (a loading dose of 4 milligrams [mg]/kilogram [kg] on Day 1, followed by a dose of 2 mg/kg on Day 1 of Week 2 and weekly thereafter) and lapatinib (1,000 mg PO daily, lapatinib 1000 mg daily, reduced to 750 mg daily during the 2-week run-in period and FEC, then increased to 1000 mg during paclitaxel (protocol amendment 3, as above).  Trastuzumab arm (randomized N = 33): trastuzumab alone (a loading dose of 4 milligrams [mg]/kilogram [kg] on Day 1, followed by a dose of 2 mg/kg on Day 1 of Week 2 and weekly thereafter)  Lapatinib arm (randomised N = 34): lapatinib alone (1250 mg orally [PO] once daily [QD]). Participants were treated with lapatinib in a 2-week run-in period  On Day 14, a second core needle biopsy was performed, followed by initiation of chemotherapy with 2 combination regimens of 4 cycles each (1 cycle=3 weeks): FEC75 (5-fluorouracil [5-FU] 500 mg/meters squared [m^2], epirubicin 75 mg/m^2, cyclophosphamide 500 mg/m^2 x 4 cycles on Day 1), then paclitaxel (80 mg/m^2 x 4 cycles on Day 1, Day 8, and Day 15) in combination with trastuzumab. |
| Outcomes | Primary: pCR (in breast and lymph nodes)  Secondary: Clinical Complete Response (CR plus PR) and safety |
| Notes | Study ID: LPT109096  ClinicalTrials identifier: NCT00524303  Median follow-up: not specified  78/100 participants were analyzed in ITT-evaluable (ITT-E). 23 of 33 and 26 of 33 participants randomized to intervention and control groups, respectively, were analyzed for pCR and clinical complete response, as well in ITT analysis. 31 of 33 and 32 of 33 participants randomized to intervention and control groups, respectively, were analyzed for safety. |

**Martin 2017**

| Methods | Accrual time: July 2009 to Oct 2011  Multicentre, randomized, double-blind, placebo-controlled, phase 3 trial.  Baseline comparability: balanced |
| --- | --- |
| Participants | 2840 female enrolled  Median age: Neratinib arm: 52 (45-59), Placebo arm: 52 (45-60)  Diagnosis: trastuzumab-based adjuvant therapy in women with HER2-positive early-stage breast cancer.  Inclusion criteria: Been treated for early breast cancer with the standard of care duration of trastuzumab. Could have been treated neoadjuvantly but have not reached pathologic complete response.aged 18 years or older (or ≥20 years in Japan, based on a Japan-specific protocol amendment [number one; Aug 3, 2009] introduced at the request of the Japanese regulatory authority) and had locally confirmed invasive HER2-positive breast cancer stage 1-3(amended to 2-3 on Feb 25, 2010 [amendment three];appendix p 5) without evidence of recurrence. HER2 status was subsequently confirmed centrally (HER2 amplification defined as a ratio of HER2 to CEP17 of ≥2.2using PathVysion HER2 DNA dual probe [Abbott Molecular, Des Plaines, IL, USA]). Hormone receptor status had to be known before study entry and was determined locally; no protocol-specified threshold for defining hormone receptor status was set. We required clinical and radiological assessments to be negative for recurrences or metastatic disease at the time of study entry; assessments included CT, MRI, or ultrasound of the abdomen and chest if hepatic transaminases or alkaline phosphatase levels were at least twice the upper limit of normal, bone scan if symptoms of metastatic bone disease were present or alkaline phosphatase levels were at least twice the upper limit of normal, or a chest radiograph. Neoadjuvant and adjuvant trastuzumab was completed up to 2 years (amended to 1 year) before randomisation. Concurrent adjuvant endocrine therapy for hormone receptor-positive disease was recommended. Patients had to have an Eastern Cooperative Oncology Group performance status of 0 or 1, normal organ function, and a left ventricular ejection fraction within normal institutional range.  HER2+: 100% |
| Interventions | 2-arm RCT  Neratinib arm (randomized N = 1420): Patients who completed prior adjuvant trastuzumab received six 40 mg neratinib tablets (240 mg) taken orally once daily for one year  Placebo arm (randomized N = 1420): Patients who completed prior adjuvant trastuzumab received six 40 mg placebo tablets (240 mg) taken orally once daily for one year |
| Outcomes | Primary: invasive disease-free survival (iDFS, time from randomization to the first occurrence of the following disease-free survival events: (a) Invasive ipsilateral breast tumor recurrence (b) Invasive contralateral breast cancer(c) Local/regional invasive recurrence (d) Distant recurrence (e) Death from any cause.)  Secondary: disease-free survival (defined as the time from randomization to the first occurrence of a disease-free survival event or ductal carcinoma in situ events), time to distant recurrence(TTDR) (defined as time from randomisation to the date of the first distant recurrence or death from breast cancer), distant disease-free survival (DDFS)(defined as time from randomisation to the first occurrence of distant recurrence or death from any cause), cumulative incidence of CNS recurrences (defined as time from randomisation to CNS recurrence as first distant recurrence-either isolated CNS metastases or those diagnosed concurrently with other sites of metastatic disease), overall survival (defined as time from randomisation to death), and safety |
| Notes | Study ID: ExteNET  Other publications: Chan 2016  ClinicalTrials identifier: NCT00878709  Median follow-up: 5.2 years   - The study was originally designed to enroll 3850 patients, In October 2011, enrolment was stopped after 2842 patients were randomly assigned and follow-up truncated to 2 years. - Patients were analyzed according to intention to treat analysis for iDFS and DFS. Patients analyzed for iDFS and DFS according to amended ITT: neratinib arm (n=938), placebo arm (n=935). Patients analyzed for safety according to safety population: neratinib arm (n=1408), placebo arm (n=1408). - The assessment of OS has not been reported because the analysis of it was planned after 248 events. - Treatment discontinuation because of treatment-emergent adverse events occurred in 388 (28%) patients in the neratinib group and 76 (5%) patients in the placebo group. - Protocol Amendment: 1.ExteNET was modified several times:1. Patients with lymph node-negative lymph nodes were initially admitted, but later were restricted to lymph node-positive patients with high-risk conditions referring to Global Protocol Amendment 3 (25-Feb-2010, Pfizer) . 2. at first, patients within 2 years of trastuzumab treatment met the enrollment criteria; after that, the conditions are limited to 1 year referring to Global Protocol Amendment 9 (14-Oct-2011, Pfizer). 3.The follow-up time was changed from 5 years to 2 years and the analysis was changed from event trigger to time trigger. In order to restore the original design, the [protocol](http://dict.youdao.com/w/protocol/#keyfrom=E2Ctranslation) was revised in 2014 and the follow-up time was extended to 5 years after regaining consent from 2117 (75%) of 2840 patients referring to Global Protocol Amendment 13 (Oct-2013, Puma Biotechnology) . |

**Piccart-Gebhart 2016**

| Methods | Accrual time: June 2007 to July 2011  International, intergroup, open-label, phase III randomized trial  Baseline comparability: balanced |
| --- | --- |
| Participants | 8381 patients ( female and male) enrolled  Median age: Trastuzumab plus lapatinib arm: 51 (22-80), Trastuzumab followed by lapatinib arm: 51 (22-80), Trastuzumab arm: 51 (18-80)  Diagnosis: histologically confirmed, completely excised invasive nonmetastatic HER2-positive breast cancer  Inclusion criteria:  1. Age ≥18 years  2. Eastern Cooperative Oncology Group (ECOG) performance status ≤1;  3. Non-metastatic operable primary invasive adenocarcinoma of the breast fulfilling the following:  a. Histologically confirmed;  b. Adequately excised (exceptions: patients who have 'non-resectable' deep margin invasion are eligible provided they have had or will receive radiotherapy encompassing the region concerned; patients with histologically documented infiltration of the skin (pT4) are eligible provided they have undergone or will receive radiotherapy encompassing the tumor bed);  c. Axilla dissected; sentinel node sampling is allowed, provided that axillary dissection follows confirmation of a positive sentinel node; sentinel node sampling alone is NOT acceptable after neoadjuvant chemotherapy (in patients receiving neoadjuvant chemotherapy lymph node status will be considered unknown, regardless of the results of post-chemotherapy axillary dissection);  d. Axillary node positive patient OR node-negative patient with a tumor greater than or equal to 1.0 cm in greatest diameter. For clarification, isolated tumor cells (ITC) are considered pN0 and micrometastases are considered pN1.  4. Known hormone receptor status (ER/PgR or ER alone);  5. For Designs 1 and 2: Patient must have received at least four cycles of an approved anthracycline-based (neo-) adjuvant chemotherapy regimen or listed as an exception in Table 5.  For Design 1: Randomization must be performed no longer than 12 weeks from Day 1 of the last chemotherapy cycle after obtaining a post-chemotherapy LVEF ≥50. Study treatment must start no more than 14 days after randomization.  For Design 2: Randomization must be performed no longer than 6 weeks from Day 1 of the last anthracycline-containing chemotherapy cycle after obtaining a post-anthracycline chemotherapy LVEF ≥50. Study treatment must start no more than 14 days after randomization and must be concurrent with taxanes.  For Design 2B: Randomization must be performed no longer than 8 weeks from definitive surgery. Non-anthracycline platinum-containing regimen (docetaxel and carboplatin) and study treatment must start concomitantly and no more than 14 days after randomization.  6. Baseline LVEF ≥50% measured by echocardiography or MUGA scan: For Design 1 and Design 2 - after completion of all anthracycline-based (neo-) adjuvant chemotherapy and prior to the targeted therapy (ies); for Design 2B - prior to targeted therapy (ies) and chemotherapy (docetaxel and carboplatin).  7. Overexpression and/or amplification of HER2 in the invasive component of a primary tumor (in case of neoadjuvant treatment, tissue sample used for HER2 testing should be collected before neoadjuvant treatment starts), according to one of the following definitions [Wolff, 2007] and confirmed by central laboratory prior to randomization:  – 3+ overexpression by IHC (>30% of invasive tumor cells);  – 2+ or 3+ (in 30% or less neoplastic cells) overexpression by IHC AND in situ hybridization (FISH/CISH) test demonstrating HER2 gene amplification;  – HER2 gene amplification by FISH/CISH (>6 HER2 gene copies per nucleus, or a FISH ratio [HER2 gene copies to chromosome 17 signals] of >2.2.)  Patients with a negative or equivocal overall result (FISH test ratio of ≤2.2, ≤6.0 HER2 gene copies per nucleus) and staining scores of 0,1+, 2+ or 3+ (in 30% or less neoplastic cells) by IHC are not eligible for participation in the trial.  Equivocal local results may be submitted for a final determination by the central laboratory.  8. Completion of all necessary baseline laboratory and radiological investigations (see Section 6.1).  9. Signed written informed consent (approved by an Independent Ethics Committee (IEC) and obtained prior to any study specific screening procedures).  HER2+: 100% |
| Interventions | 4-arm RCT  Trastuzumab and lapatinib arm (randomized N = 2093): participants (par.) received treatment per one of three designs. D1: oral lapatinib (OL) 1000 milligrams (mg) daily with trastuzumab (8 milligrams per kilogram [mg/kg] intravenous [IV] loading dose [LD], followed by 6 mg/kg IV every 3 weeks [E3W]) for 52 weeks (wks). D2: OL 750 mg daily plus weekly trastuzumab (4 mg/kg LD, followed by 2 mg/kg IV) concomitantly (conc.) with wkly paclitaxel (pac) 80 mg per squared meter (mg/m^2) IV or docetaxel (doc) 75 mg/m^2 IV E3W for 12 wks. After completion of pac or doc, par. received OL at an increased dose of 1000 mg daily in combination with trastuzumab (6 mg/kg without a LD) E3W for 40 wks. D2B: OL 750 mg plus wkly tratuzumab (4 mg/kg IV LD, followed by 2 mg/kg IV wkly) conc. with doc 75 mg/m^2 E3W and carboplatin (carb) AUC6 IV for 18 wks. After completion of doc and carb, par. received trastuzumab E3W (6 mg/kg without a LD) plus OL 1000 mg daily for 34 wks.  Trastuzumab followed by lapatinib arm (randomized N = 2091): participants received treatment per one of the following three designs. Design 1: weekly tras for 12 weeks (4 mg/kg IV loading dose, followed by 2 mg/kg IV weekly), followed by a 6-week washout period, followed by oral lap 1500 mg daily for 34 weeks. Design 2: weekly tras (4 mg/kg IV loading dose, followed by 2 mg/kg IV) concomitantly with weekly paclitaxel 80 mg/m^2 IV or docetaxel 75 mg/m^2 IV every 3 weeks for 12 weeks, followed by a 6-week washout period, followed by oral lap 1500 mg daily for 34 weeks. Design 2B: weekly trastuzumab (4 mg/kg IV loading dose, followed by 2 mg/kg IV weekly) concomitantly with docetaxel 75 mg/m^2 every 3 weeks and carboplatin AUC6 IV for 18 weeks, followed by a 6-week washout period, followed by oral lap 1500 mg daily for 28 weeks  Trastuzumab arm (randomized N = 2097): participants received treatment per one of the following three designs. Design 1: trastuzumab 8 mg/kg IV LD, followed by 6 mg/kg IV every 3 weeks for 52 weeks. Design 2: weekly trastuzumab (4 mg/kg IV LD, followed by 2 mg/kg IV weekly) concomitantly with weekly paclitaxel 80 mg/m^2 IV or docetaxel 75 mg/m^2 IV every 3 weeks, for 12 weeks. After completion of paclitaxel or docetaxel, participants received trastuzumab (6 mg/kg without a LD every 3 weeks for 40 weeks. Design 2B: weekly trastuzumab (4 mg/kg IV LD, followed by 2 mg/kg IV weekly) concomitantly with docetaxel 75 mg/m^2 every 3 weeks and carboplatin AUC6 IV, for 18 weeks. After completion of docetaxel and carboplatin, participants received trastuzumab every 3 weeks (6 mg/kg without a LD) for 34 weeks.  Lapatinib arm (randomized N = 2100): participants received treatment per one of the following three designs. Design 1: oral lap 1500 mg daily for 52 weeks. Design 2: oral lap 750 mg daily concomitantly with weekly paclitaxel 80 mg/m^2 IV or docetaxel 75 mg/m^2 IV every 3 weeks, for 12 weeks. After completion of paclitaxel or docetaxel, participants received oral lap at an increased dose of 1500 mg daily for 40 weeks. Design 2B: oral lap 750 mg daily concomitantly with docetaxel 75 mg/m^2 every 3 weeks and carboplatin AUC6 IV, for 18 weeks. After completion of docetaxel and carboplatin, oral lap was given at an increased dose of 1500 mg for 34 weeks. |
| Outcomes | Primary: DFS (disease-free survival: defined as time from randomization to recurrence of invasive breast cancer at local, regional, or distant sites; contralateral invasive breast cancer; second non-breast malignancy; or death as a result of any cause, whichever occurred first. This is the same as invasive disease-free survival per subpopulation treatment effect pattern plot (STEPP))  Secondary: OS, safety in general, cardiac safety, TTR (time to recurrence: from randomization until the date of the first occurrence of a disease recurrence), TTDR (time to distant recurrence: from randomization until the date of the first occurrence of distant recurrence), and time to first brain metastasis. |
| Notes | Study ID: ALTTO  Other publications: Moreno-Aspitia 2017  ClinicalTrials identifier: NCT00490139  Median follow-up: 6.9 years   - The trial had a large proportion of low-stage disease (T1 tumors and node-negative disease) - In 2011, due to futility to demonstrate non-inferiority of L versus T, the L arm was closed, and patients free of disease were offered adjuvant T. - Patients were analyzed according to intention to treat analysis for DFS and OS. Patients were analyzed according to safety Population (including all randomly assigned patients who received at least one dose of anti-HER2 therapy) analysis for safety: 2061/2093 women received trastuzumab plus lapatinib, 2076/2091 women received lapatinib sequential trastuzumab treatment and 2076/2097 women received trastuzumab alone. |

**Robidoux 2013**

| Methods | Accrual time: July 2007 to June 2011  Open-label randomized phase 3 trial  Baseline comparability: balanced |
| --- | --- |
| Participants | 529 female enrolled  Median age: not reported  Diagnosis: HER2-positive operable breast cancer.  Inclusion criteria: women aged 18 years or older with an ECOG performance status of 0 or 1 with operable HER2-positive breast cancer were eligible. breast tumor at least 2 cm or larger by palpation; clinical stage T2 to T3, N0 to N2a disease; no evidence of metastases; no history of previous invasive breast cancer; diagnosis by core needle biopsy; tumor with HER2 gene amplification by fluorescent in situ hybridisation (FISH) or chromogenic in situ hybridisation(CISH), or a strong positive (3+) staining score by immunohistochemistry; left ventricular ejection fraction(LVEF) assessment by multiple-gated acquisition scan or echocardiogram, of 50% or higher, regardless of the lower limit of normal of the assessing facility; no active cardiac disease or history of documented myocardial infarction, congestive heart failure, or cardiomyopathy.  HER2+: 100% |
| Interventions | 3-arm RCT  AC then paclitaxel + trastuzumab + lapatinib arm (randomized N = 174): AC followed by paclitaxel plus trastuzumab plus lapatinib (trastuzumab 4 mg/kg IV followed by 2 mg/kg IV weekly beginning on day 1 of the first paclitaxel cycle until 1-7 days before surgery plus lapatinib 750 mg PO daily beginning on day 1 of the first paclitaxel cycle until 1 day before surgery)  AC then paclitaxel + trastuzumab arm (randomized N = 181): AC then paclitaxel + trastuzumab (first dose: 4 mg/kg IV, subsequent doses: 2 mg/kg IV weekly beginning on day 1 of the first paclitaxel cycle until 1-7 days before surgery)  AC then paclitaxel + lapatinib arm (randomized N = 174): lapatinib 1250 mg PO daily beginning on day 1 of the first paclitaxel cycle until 1 day before surgery  AC followed by paclitaxel: four cycles of doxorubicin 60 mg/m2 and cyclophosphamide 600 mg/m2 intravenously on day 1 every 3 weeks, followed by four cycles of paclitaxel 80 mg/m2 intravenously on days 1, 8, and 15, every 4 weeks. |
| Outcomes | Primary: pathological complete response (pCR) in the breast  Secondary: pathological complete response (pCR) in the breast and nodes, clinical complete response, severe cardiac events (defined as definite or probable cardiac death, or New York Heart Association (NYHA) class III or IV congestive heart failure), non-cardiac toxic effects, OS and RFI (recurrence-free interval). |
| Notes | Study ID: NSABP B-41  Other publications: Robidoux 2016  ClinicalTrials identifier: NCT00486668  Median follow-up: 5 years   - 12 patients withdrew consent: three from the trastuzumab group, four from the lapatinib group, and five from the combination group; seven patients withdrew before assessment of pathological response, five after assessment of pathological response. Another eight patients were ineligible: three from the trastuzumab group, two from the lapatinib group, and three from the combination group. - June 10, 2008, we reduced the starting dose of lapatinib to 1250 mg in the lapatinib group and to 750 mg in the combination group because of the excessive number of patients developing diarrhea reported in other trials using the initial doses used in NSABP B-41. - 7 (4%) patients in the lapatinib group discontinued lapatinib and crossed over to trastuzumab. 26 (15%) patients in the trastuzumab group, 38 (24%) in the lapatinib group, and 43 (26%) in the combination group discontinued neoadjuvant therapy because of adverse events. - All randomized participants were analyzed according to ITT analyses for OS and RFI. 171/174 women received trastuzumab plus pertuzumab, 177/181 women received trastuzumab alone for pCR in the breast. 171/174 and 176/181 for pCR in the breast and nodes. 164/174 and 167/181 for clinical response. 173/174 and 167/178 for safety. |

**Von Minckwitz 2017**

| Methods | Accrual time: November 2011 to August 2013  Prospective, two-group, randomized, multicenter, multinational, double-blind, placebo-controlled Phase 3 trial  Baseline comparability: baseline characteristics of the patients were balanced between the two groups, with 64% having HR-positive disease and 36% having HR-negative disease. |
| --- | --- |
| Participants | 4805 patients (female and male) enrolled  Median age: not reported  Diagnosis: non-metastatic, adequately excised, histologically confirmed invasive HER2-positive breast cancer.  Inclusion criteria: Patients with synchronous bilateral invasive disease were eligible if both lesions were HER2-positive. Eligible patients had to have either node-positive disease or node-negative disease with a tumor diameter greater than 1.0 cm. Patients with node-negative tumors between 0.5 and 1.0 cm in diameter were initially eligible if at least one of the following high-risk features was present: histologic or nuclear grade 3, negativity for estrogen and progesterone receptors, or age younger than 35 years.Under a protocol amendment that was added after 3655 patients had undergone randomization, patients with node-negative disease were no longer eligible for enrollment, in order to enroll a patient population with the nodal-status distribution that had been anticipated when the trial was designed. The interval between definitive breast surgery and the first chemotherapy dose had to be within 8 weeks. The baseline left ventricular ejection fraction had to be at least 55%.  HER2+: 100% |
| Interventions | 2-arm RCT  Trastuzumab and pertuzumab arm (randomized N = 2400): participants received pertuzumab (840 mg loading dose, then 420 mg) and trastuzumab (8 mg/kg loading dose, then 6 mg/kg) IV Q3W for 1 year (maximum 18 cycles) in combination with 1 of the following IV chemotherapy regimen (anthracycline-based or nonanthracycline-based) per Investigator's choice.  Trastuzumab and placebo arm (randomized N = 2405): participants received placebo matched to pertuzumab IV Q3W and trastuzumab (8 mg/kg loading dose, then 6 mg/kg) IV Q3W for 1 year (maximum 18 cycles) in combination with 1 of the following IV chemotherapy regimen (anthracycline-based or nonanthracycline-based) per Investigator's choice.  Investigator's choice: 1) 3-4 cycles (Q3W) of 5-fluorouracil 500-600 mg/m^2 + epirubicin 90-120 mg/m^2 or doxorubicin 50 mg/m^2 + cyclophosphamide 500-600 mg/m^2 followed by either 3-4 cycles of docetaxel Q3W (100 mg/m^2 for 3 cycles, 75 mg/m^2 in first cycle and 100 mg/m^2 in subsequent cycles, or 75 mg/m^2 for 4 cycles) or 12 cycles of paclitaxel 80 mg/m^2 QW; 2) 4 cycles (Q3W) of doxorubicin 60 mg/m^2 or epirubicin 90-120 mg/m^2 + cyclophosphamide 500-600 mg/m^2 followed by either 3-4 cycles of docetaxel Q3W or 12 cycles of paclitaxel QW (as described in Option 1); 3) 6 cycles (Q3W) of docetaxel 75 mg/m^2 + carboplatin area under the curve (AUC) 6 (up to 900 mg). |
| Outcomes | Primary: iDFS (invasive disease-free survival excluding second primary non-breast cancers as events is not the same as iDFS defined by Hudis et al.)  Secondary: OS (overall survival), iDFS (invasive disease-free survival include second primary non-breast cancer, per the STEEP definition), DFS (disease-free survival include second primary non-breast cancers or contralateral or ipsilateral ductal carcinoma in-situ as an event), relapse-free interval, distant relapse-free interval, safety, and health-related quality of life. |
| Notes | Study ID: APIHINITY  ClinicalTrials identifier: NCT01358877  Median follow-up: 45.4 months  4804 of 4805 randomized participants were analyzed in ITT analyses for DFS, iDFS and OS. One patient excluded from the ITT population because of her falsification of personal Information. 2364/2400 women in the combination group, 2405/2405 women in the trastuzumab group were analyzed for safety. |

# Appendix 3. Risk of bias assessment

For each risk of bias domain, we assigned a judgment according to the risk of bias tool of The Cochrane Collaboration* as follows: 1) Low: indicated a low risk of bias. 2) High: indicated a high risk of bias. 3) Unclear: indicated an unclear or unknown.

***Selection bias***

- Random sequence generation

Low risk: if the allocation sequence was generated from a random number table, by computer or by minimization. And examined if there were imbalances across the arms.

High risk: if the study used a non-random approach.

Unclear: if it was stated that the trial was randomized but the method was not described.

- Allocation concealment

Low risk: if the following methods were used to conceal allocation: central allocation, or sequentially numbered, opaque, sealed envelopes.

High risk: if the study used an open random allocation schedule or any other explicitly unconcealed procedure.

Unclear: if the method of allocation concealment was not described.

***Performance bias***

- Blinding of participants and personnel

Low risk: If blinding of participants and key study personnel ensured, or unlikely that the outcome could have been influenced by lack of blinding.

High risk: If no blinding of outcome assessment or the outcome was likely to be influenced by lack of blinding.

Unclear risk: If the study did not address this outcome or did not have sufficient information to permit judgment of “Low risk” or “High risk”.

***Detection bias***

- Blinding of outcome assessors

*OS and outcomes other than OS (EFS/iDFS, ORR, pCR, and safety) was considered separately because it is unlikely that assessment of OS would be affected by lack of blinding.*

Low risk: Blinding of outcome assessment ensured, or unlikely that the outcome could have been influenced by lack of blinding (OS).

High risk: No blinding of outcome assessment and the outcome measurement was likely to be influenced by lack of blinding.

Unclear risk: if the study did not address this outcome or did not have sufficient information to permit judgment of “Low risk” or “High risk”.

***Attrition bias***

- Incomplete outcome data

We examined if there was an imbalance in numbers or reasons for missing data across intervention groups and if the analysis was carried out based on intention to treat.

For time-to-event outcomes (OS, EFS/iDFS):

Low risk: if the study performed the analysis according to intention-to-treat (ITT).

High risk: if the study performed the analysis by means of per-protocol.

Unclear risk: if the study performed the analysis according to modified intention-to-treat (mITT).

For dichotomous outcomes (ORR, pCR, and safety):

Low risk: if the percentage of missing data was less than 10%.

High risk: if the percentage of missing data was greater than 15%.

Unclear risk: if the percentage of missing data was 10% to 15%.

***Reporting bias***

- Selective outcome reporting

Low risk: if the study protocol was available and all of the study’s pre-specified (primary and secondary) outcomes that are of interest in the review have been reported.

High risk: if one or more outcomes of interest in the review are reported incompletely so that they cannot be entered in a meta-analysis;

Unclear risk: if there was no sufficient information to permit judgment of ‘Low risk’ or ‘High risk’.

***Other bias***

We examined if there were any other potential sources of bias.

The final risk of bias assessments as the table below

**Risk of bias assessment**

| **Study** | **Random sequence generation** | **Allocation concealment** | **Blinding of participants and personnel** | **Blinding of outcome assessment (OS)** | **Blinding of outcome assessors (outcomes other than OS)** | **Incomplete outcome data (time-to-event outcomes)** | **Incomplete outcome data (dichotomous outcomes)** | **Selective reporting** | **Other bias** |
| --- | --- | --- | --- | --- | --- | --- | --- | --- | --- |
| Gianni 2016 | low risk | low risk | high risk | unclear | low risk | low risk | low risk | low risk | low risk |
| Von Minckwitz 2017 | low risk | unclear | low risk | low risk | unclear | low risk | low risk | low risk | unclear |
| Piccart-Gebhart2016 | low risk | unclear | high risk | low risk | unclear | low risk | low risk | low risk | unclear |
| Carey 2016 | unclear | unclear | high risk | low risk | unclear | unclear | low risk | low risk | low risk |
| De Azambuja 2014 | low risk | low risk | high risk | low risk | low risk | low risk | low risk | low risk | low risk |
| Guarneri 2012 | unclear | low risk | high risk | unclear | low risk | unclear | unclear | low risk | unclear |
| Holmes 2013 | unclear | unclear risk | high risk | unclear | unclear | unclear | high risk | low risk | low risk |
| Robidoux 2013 | low risk | low risk | high risk | low risk | unclear | low risk | low risk | low risk | unclear |
| Bonnefoi 2014 | low risk | low risk | high risk | unclear | low risk | unclear risk | low risk | low risk | low risk |
| Martin 2017 | low risk | low risk | low risk | low risk | low risk | low risk | low risk | low risk | unclear |

*Reference: Higgins J P T, Green S (2011) Cochrane handbook for systematic reviews of interventions. Version 5.1. 0 [updated March 2011]. The Cochrane Collaboration. https://training.cochrane.org/handbook. Accessed 10 December 2018
