# Supplementary material for: Dual HER2 Blockade versus a Single Agent in Trastuzumab-Containing Regimens for HER2-Positive Early Breast Cancer: A Systematic Review and Meta-Analysis of Randomized Controlled Trials
Source: J Oncol. 2020 Mar 16;2020:5169278. doi: 10.1155/2020/5169278 (PMC7102417; doi:10.1155/2020/5169278)
Supplement: Supplementary Materials — Additional file 1: Appendix 1: definitions of outcomes. Appendix 2: characteristics of included studies. Appendix 3: risk of bias assessment. Additional file 2: Figure S1: forest plots for all outcomes. Figure S2: sensitivity analyses. Figure S3: publication bias. [file 5169278.f1.zip › 5169278.f1/Additional file 2.doc]

**Supplementary material (Additional file 2)**

**Brief Summary**

**Outcomes including event-free survival/invasive disease-free survival(EFS/iDFS), overall survival(OS), overall response rate (ORR), pathological complete response (pCR) rate in breast and axillary LNs, cardiac toxicity and other toxicities (diarrhoea, hepatic toxicity, skin disorder, neutropenia, febrile neutropenia, nausea and vomiting, fatigue) were considered in our meta-analysis.**

**Figure S1, which contains the forest plots for all the above outcomes. Following subgroup analyses were performed: treatment setting (neoadjuvant or adjuvant setting), dual anti-HER2 regimen (tastuzumab plus lapatinib, trastuzumab plus pertuzumab or trastuzumab plus neratinib), chemotherapy regimen (taxane-containing but non-anthracycline, anthracycline plus taxane, or other), Lymph Node (LN) status, and hormone receptor (HR) status.**

| **Outcomes** | | **Subgroup** | | | | |
| --- | --- | --- | --- | --- | --- | --- |
|  |  | **Treatment Setting** | **Dual HER2 Blockade Regimen** | **HR Status** | **LN Status** | **Chemotherapy Regimen** |
| **OS** | | **Yes** | **Yes** | **/** | **/** | **/** |
| **EFS/iDFS** | | **Yes** | **Yes** | **Yes** | **Yes** | **/** |
| **ORR** | | **/** | **Yes** | **/** | **/** | **/** |
| **pCR** | | **/** | **Yes** | **Yes** | **/** | **/** |
| **Cardiac Toxicity** | **CHF** | **Yes** | **/** | **/** | **/** |  |
|  | **LVEF Decline** |  | **/** | **/** | **/** |  |
| **CHF** | | **Yes** | **/** | **/** | **/** | **Yes** |
| **LVEF Decline** | | **Yes** | **/** | **/** | **/** | **Yes** |
| **Other Toxicities** | | **Yes** | **/** | **/** | **/** | **Yes** |

**Figure S2, which contains sensitivity analyses of OS, EFS/iDFS, ORR, pCR using the leave-one-out procedure. The results were stable.**

**Figure S3, which contains publication bias of OS and EFS/iDFS. No evidence of publication bias was observed.**

**Abbreviations**

**T	Trastuzumab	L	Lapatinib**

**P	Pertuzumab	N	Neratinib**

**HR+	Hormone receptor-positive	HR-	Hormone receptor-negative**

**LN+	Lymph node-positive	LN-	Lymph node-negative**

**Figure S1. Forest plots for all outcomes	4**

Analysis 1.1. Comparison 1 Efficacy of trastuzumab-containing dual anti-HER2 therapy, Outcome 1 Overall survival - all studies	4

Analysis 1.2. Comparison 1 Efficacy of trastuzumab-containing dual anti-HER2 therapy, Outcome 2 Overall survival - stratified by treatment setting	4

Analysis 1.3. Comparison 1 Efficacy of trastuzumab-containing dual anti-HER2 therapy, Outcome 3 Overall survival - stratified by type of dual HER2 blockade regimen	4

Analysis 1.4. Comparison 1 Efficacy of trastuzumab-containing dual anti-HER2 therapy, Outcome 4 Event-free survival/invasive disease-free survival - all studies	5

Analysis 1.5. Comparison 1 Efficacy of trastuzumab-containing dual anti-HER2 therapy, Outcome 5 Event-free survival/invasive disease-free survival - stratified by treatment setting	5

Analysis 1.6. Comparison 1 Efficacy of trastuzumab-containing dual anti-HER2 therapy, Outcome 6 Event-free survival/invasive disease-free survival - stratified by type of dual HER2 blockade regimen	6

Analysis 1.7. Comparison 1 Efficacy of trastuzumab-containing dual anti-HER2 therapy, Outcome 7 Event-free survival/invasive disease-free survival - stratified by hormone receptor status	6

Analysis 1.8. Comparison 1 Efficacy of trastuzumab-containing dual anti-HER2 therapy, Outcome 8 Event-free survival/invasive disease-free survival - stratified by lymph node status	7

Analysis 1.9. Comparison 1 Efficacy of trastuzumab-containing dual anti-HER2 therapy, Outcome 9 Overall response rate - all studies	7

Analysis 1.10. Comparison 1 Efficacy of trastuzumab-containing dual anti-HER2 therapy, Outcome 10 Overall response rate - stratified by type of dual HER2 blockade regimen	7

Analysis 1.11. Comparison 1 Efficacy of trastuzumab-containing dual anti-HER2 therapy, Outcome 11 Pathological complete response - all studies	8

Analysis 1.12. Comparison 1 Efficacy of trastuzumab-containing dual anti-HER2 therapy, Outcome 12 Pathological complete response - stratified by type of dual HER2 blockade regimen	8

Analysis 1.13. Comparison 1 Efficacy of trastuzumab-containing dual anti-HER2 therapy, Outcome 13 Pathological complete response - stratified by hormone receptor status	9

Analysis 2.1. Comparison 2 Cardiac toxicity of trastuzumab-containing dual anti-HER2 therapy, Outcome 1 Cardiac toxicity - all studies	9

Analysis 2.2. Comparison 2 Cardiac toxicity of trastuzumab-containing dual anti-HER2 therapy, Outcome 2 Cardiac toxicity - stratified by treatment setting	10

Analysis 2.3. Comparison 2 Cardiac toxicity of trastuzumab-containing dual anti-HER2 therapy, Outcome 2 Cardiac toxicity - stratified by congestive heart failure and left ventricular ejection fraction decline	10

Analysis 2.4. Comparison 2 Cardiac toxicity of trastuzumab-containing dual anti-HER2 therapy, Outcome 3 Congestive heart failure - stratified by type of dual HER2 blockade regimen	11

Analysis 2.5. Comparison 2 Cardiac toxicity of trastuzumab-containing dual anti-HER2 therapy, Outcome 4 Congestive heart failure - stratified by type of chemotherapy regimen	11

Analysis 2.6. Comparison 2 Cardiac toxicity of trastuzumab-containing dual anti-HER2 therapy, Outcome 5 Left ventricular ejection fraction decline - stratified by type of dual HER2 blockade regimen	12

Analysis 2.7. Comparison 2 Cardiac toxicity of trastuzumab-containing dual anti-HER2 therapy, Outcome 6 Left ventricular ejection fraction decline - stratified by type of chemotherapy regimen	12

Analysis 3.1. Comparison 2 Other toxicities of trastuzumab-containing dual anti-HER2 therapy, Outcome 1 Diarrhoea- all studies	13

Analysis 3.2. Comparison 2 Other toxicities of trastuzumab-containing dual anti-HER2 therapy, Outcome 2 Diarrhoea - stratified by type of dual HER2 blockade regimen	13

Analysis 3.3. Comparison 2 Other toxicities of trastuzumab-containing dual anti-HER2 therapy, Outcome 3 Diarrhoea - stratified by type of chemotherapy regimen	14

Analysis 3.4. Comparison 2 Other toxicities of trastuzumab-containing dual anti-HER2 therapy, Outcome 4 Hepatic toxicity - all studies	14

Analysis 3.5. Comparison 2 Other toxicities of trastuzumab-containing dual anti-HER2 therapy, Outcome 5 Hepatic toxicity - stratified by type of dual HER2 blockade regimen	15

Analysis 3.6. Comparison 2 Other toxicities of trastuzumab-containing dual anti-HER2 therapy, Outcome 6 Hepatic toxicity - stratified by type of chemotherapy regimen	16

Analysis 3.7. Comparison 2 Other toxicities of trastuzumab-containing dual anti-HER2 therapy, Outcome 7 Skin disorder - all studies	16

Analysis 3.8. Comparison 2 Other toxicities of trastuzumab-containing dual anti-HER2 therapy, Outcome 8 Skin disorder - stratified by type of dual HER2 blockade regimen	17

Analysis 3.9. Comparison 2 Other toxicities of trastuzumab-containing dual anti-HER2 therapy, Outcome 9 Skin disorder - stratified by type of chemotherapy regimen	18

Analysis 3.10. Comparison 2 Other toxicities of trastuzumab-containing dual anti-HER2 therapy, Outcome 10 Neutropenia - all studies	18

Analysis 3.11. Comparison 2 Other toxicities of trastuzumab-containing dual anti-HER2 therapy, Outcome 11 Neutropenia - stratified by type of dual HER2 blockade regimen	19

Analysis 3.12. Comparison 2 Other toxicities of trastuzumab-containing dual anti-HER2 therapy, Outcome 12 Neutropenia - stratified by type of chemotherapy regimen	19

Analysis 3.13. Comparison 2 Other toxicities of trastuzumab-containing dual anti-HER2 therapy, Outcome 13 Febrile neutropenia - all studies	20

Analysis 3.14. Comparison 2 Other toxicities of trastuzumab-containing dual anti-HER2 therapy, Outcome 14 Febrile neutropenia - stratified by type of dual HER2 blockade regimen	20

Analysis 3.15. Comparison 2 Other toxicities of trastuzumab-containing dual anti-HER2 therapy, Outcome 15 Febrile neutropenia - stratified by type of chemotherapy regimen	21

Analysis 3.16. Comparison 2 Other toxicities of trastuzumab-containing dual anti-HER2 therapy, Outcome 16 Nausea and vomiting - all studies	21

Analysis 3.17. Comparison 2 Other toxicities of trastuzumab-containing dual anti-HER2 therapy, Outcome 17 Nausea and vomiting - stratified by type of dual HER2 blockade regimen	22

Analysis 3.18. Comparison 2 Other toxicities of trastuzumab-containing dual anti-HER2 therapy, Outcome 18 Nausea and vomiting - stratified by type of chemotherapy regimen	22

Analysis 3.19. Comparison 2 Other toxicities of trastuzumab-containing dual anti-HER2 therapy, Outcome 19 Fatigue - all studies	23

Analysis 3.20. Comparison 2 Other toxicities of trastuzumab-containing dual anti-HER2 therapy, Outcome 10 Fatigue - stratified by type of dual HER2 blockade regimen	23

Analysis 3.21. Comparison 2 Other toxicities of trastuzumab-containing dual anti-HER2 therapy, Outcome 21 Fatigue - stratified by type of chemotherapy regimen	24

Figure S2. Sensitivity analyses	25

Analysis 4.1. Comparison 4 Sensitivity analysis - by leave-one-out procedure, Outcome 1 Overall survival	25

Analysis 4.2. Comparison 4 Sensitivity analysis - by leave-one-out procedure, Outcome 2 Event-free survival/invasive disease-free survival	25

Analysis 4.3. Comparison 4 Sensitivity analysis - by leave-one-out procedure, Outcome 3 Overall response rate	26

Analysis 4.4. Comparison 4 Sensitivity analysis - by leave-one-out procedure, Outcome 4 Pathological complete response	26

Analysis 4.5. Comparison 4 Sensitivity analysis - by leave-one-out procedure, Outcome 5 Cardiac toxicity	27

Figure S3. Publication bias	27

Funnel plot 1: Plot for Overall survival (OS).	27

Funnel plot 2: Plot for Event-free survival/invasive disease-free survival (EFS/iDFS).	28

# Figure S1. Forest plots for all outcomes

## Analysis 1.1. Comparison 1 Efficacy of trastuzumab-containing dual anti-HER2 therapy, Outcome 1 Overall survival - all studies

## Analysis 1.2. Comparison 1 Efficacy of trastuzumab-containing dual anti-HER2 therapy, Outcome 2 Overall survival - stratified by treatment setting

## Analysis 1.3. Comparison 1 Efficacy of trastuzumab-containing dual anti-HER2 therapy, Outcome 3 Overall survival - stratified by type of dual HER2 blockade regimen

## Analysis 1.4. Comparison 1 Efficacy of trastuzumab-containing dual anti-HER2 therapy, Outcome 4 Event-free survival/invasive disease-free survival - all studies

## Analysis 1.5. Comparison 1 Efficacy of trastuzumab-containing dual anti-HER2 therapy, Outcome 5 Event-free survival/invasive disease-free survival - stratified by treatment setting

## Analysis 1.6. Comparison 1 Efficacy of trastuzumab-containing dual anti-HER2 therapy, Outcome 6 Event-free survival/invasive disease-free survival - stratified by type of dual HER2 blockade regimen

## Analysis 1.7. Comparison 1 Efficacy of trastuzumab-containing dual anti-HER2 therapy, Outcome 7 Event-free survival/invasive disease-free survival - stratified by hormone receptor status

## Analysis 1.8. Comparison 1 Efficacy of trastuzumab-containing dual anti-HER2 therapy, Outcome 8 Event-free survival/invasive disease-free survival - stratified by lymph node status

## Analysis 1.9. Comparison 1 Efficacy of trastuzumab-containing dual anti-HER2 therapy, Outcome 9 Overall response rate - all studies

## Analysis 1.10. Comparison 1 Efficacy of trastuzumab-containing dual anti-HER2 therapy, Outcome 10 Overall response rate - stratified by type of dual HER2 blockade regimen

## Analysis 1.11. Comparison 1 Efficacy of trastuzumab-containing dual anti-HER2 therapy, Outcome 11 Pathological complete response - all studies

## Analysis 1.12. Comparison 1 Efficacy of trastuzumab-containing dual anti-HER2 therapy, Outcome 12 Pathological complete response - stratified by type of dual HER2 blockade regimen

## Analysis 1.13. Comparison 1 Efficacy of trastuzumab-containing dual anti-HER2 therapy, Outcome 13 Pathological complete response - stratified by hormone receptor status

Analysis 2.1. Comparison 2 Cardiac toxicity of trastuzumab-containing dual anti-HER2 therapy, Outcome 1 Cardiac toxicity - all studies

Analysis 2.2. Comparison 2 Cardiac toxicity of trastuzumab-containing dual anti-HER2 therapy, Outcome 2 Cardiac toxicity - stratified by treatment setting

## Analysis 2.3. **Comparison 2 Cardiac toxicity of trastuzumab-containing dual anti-HER2 therapy, Outcome 2 Cardiac toxicity - stratified by** congestive heart failure and left ventricular ejection fraction decline

## Analysis 2.4. Comparison 2 Cardiac toxicity of trastuzumab-containing dual anti-HER2 therapy, Outcome 3 Congestive heart failure - stratified by type of dual HER2 blockade regimen

## Analysis 2.5. Comparison 2 Cardiac toxicity of trastuzumab-containing dual anti-HER2 therapy, Outcome 4 Congestive heart failure - stratified by type of chemotherapy regimen

## Analysis 2.6. Comparison 2 Cardiac toxicity of trastuzumab-containing dual anti-HER2 therapy, Outcome 5 Left ventricular ejection fraction decline - stratified by type of dual HER2 blockade regimen

## Analysis 2.7. Comparison 2 Cardiac toxicity of trastuzumab-containing dual anti-HER2 therapy, Outcome 6 Left ventricular ejection fraction decline - stratified by type of chemotherapy regimen

## Analysis 3.1. Comparison 2 Other toxicities of trastuzumab-containing dual anti-HER2 therapy, Outcome 1 Diarrhoea- all studies

## Analysis 3.2. Comparison 2 Other toxicities of trastuzumab-containing dual anti-HER2 therapy, Outcome 2 Diarrhoea - stratified by type of dual HER2 blockade regimen

## Analysis 3.3. Comparison 2 Other toxicities of trastuzumab-containing dual anti-HER2 therapy, Outcome 3 Diarrhoea - stratified by type of chemotherapy regimen

## Analysis 3.4. Comparison 2 Other toxicities of trastuzumab-containing dual anti-HER2 therapy, Outcome 4 Hepatic toxicity - all studies

## Analysis 3.5. Comparison 2 Other toxicities of trastuzumab-containing dual anti-HER2 therapy, Outcome 5 Hepatic toxicity - stratified by type of dual HER2 blockade regimen

## Analysis 3.6. Comparison 2 Other toxicities of trastuzumab-containing dual anti-HER2 therapy, Outcome 6 Hepatic toxicity - stratified by type of chemotherapy regimen

## Analysis 3.7. Comparison 2 Other toxicities of trastuzumab-containing dual anti-HER2 therapy, Outcome 7 Skin disorder - all studies

## Analysis 3.8. Comparison 2 Other toxicities of trastuzumab-containing dual anti-HER2 therapy, Outcome 8 Skin disorder - stratified by type of dual HER2 blockade regimen

## Analysis 3.9. Comparison 2 Other toxicities of trastuzumab-containing dual anti-HER2 therapy, Outcome 9 Skin disorder - stratified by type of chemotherapy regimen

## Analysis 3.10. Comparison 2 Other toxicities of trastuzumab-containing dual anti-HER2 therapy, Outcome 10 Neutropenia - all studies

## Analysis 3.11. Comparison 2 Other toxicities of trastuzumab-containing dual anti-HER2 therapy, Outcome 11 Neutropenia - stratified by type of dual HER2 blockade regimen

## Analysis 3.12. Comparison 2 Other toxicities of trastuzumab-containing dual anti-HER2 therapy, Outcome 12 Neutropenia - stratified by type of chemotherapy regimen

## Analysis 3.13. Comparison 2 Other toxicities of trastuzumab-containing dual anti-HER2 therapy, Outcome 13 Febrile neutropenia - all studies

## Analysis 3.14. Comparison 2 Other toxicities of trastuzumab-containing dual anti-HER2 therapy, Outcome 14 Febrile neutropenia - stratified by type of dual HER2 blockade regimen

## Analysis 3.15. Comparison 2 Other toxicities of trastuzumab-containing dual anti-HER2 therapy, Outcome 15 Febrile neutropenia - stratified by type of chemotherapy regimen

## Analysis 3.16. Comparison 2 Other toxicities of trastuzumab-containing dual anti-HER2 therapy, Outcome 16 Nausea and vomiting - all studies

## Analysis 3.17. Comparison 2 Other toxicities of trastuzumab-containing dual anti-HER2 therapy, Outcome 17 Nausea and vomiting - stratified by type of dual HER2 blockade regimen

## Analysis 3.18. Comparison 2 Other toxicities of trastuzumab-containing dual anti-HER2 therapy, Outcome 18 Nausea and vomiting - stratified by type of chemotherapy regimen

## Analysis 3.19. Comparison 2 Other toxicities of trastuzumab-containing dual anti-HER2 therapy, Outcome 19 Fatigue - all studies

## Analysis 3.20. Comparison 2 Other toxicities of trastuzumab-containing dual anti-HER2 therapy, Outcome 10 Fatigue - stratified by type of dual HER2 blockade regimen

## Analysis 3.21. Comparison 2 Other toxicities of trastuzumab-containing dual anti-HER2 therapy, Outcome 21 Fatigue - stratified by type of chemotherapy regimen

# Figure S2. Sensitivity analyses

## Analysis 4.1. Comparison 4 Sensitivity analysis - by leave-one-out procedure, Outcome 1 Overall survival

## Analysis 4.2. Comparison 4 Sensitivity analysis - by leave-one-out procedure, Outcome 2 Event-free survival/invasive disease-free survival

## Analysis 4.3. Comparison 4 Sensitivity analysis - by leave-one-out procedure, Outcome 3 Overall response rate

## Analysis 4.4. Comparison 4 Sensitivity analysis - by leave-one-out procedure, Outcome 4 Pathological complete response

## Analysis 4.5. Comparison 4 Sensitivity analysis - by leave-one-out procedure, Outcome 5 Cardiac toxicity

# Figure S3. Publication bias

Funnel plot 1: Plot for Overall survival (OS). The plot does not show asymmetry (Begg’s test P value = 0.327)

Funnel plot 2: Plot for Event-free survival/invasive disease-free survival (EFS/iDFS). The plot does not show asymmetry (Begg’s test P value = 0.091)
